# Normal and Extreme Wind Conditions for Power at Coastal Locations in China

**DOI:** 10.1371/journal.pone.0136876

**Published:** 2015-08-27

**Authors:** Meng Gao, Jicai Ning, Xiaoqing Wu

**Affiliations:** Yantai Institute of Coastal Zone Research, Chinese Academy of Sciences, Yantai, China; Centro de Investigacion Cientifica y Educacion Superior de Ensenada, MEXICO

## Abstract

In this paper, the normal and extreme wind conditions for power at 12 coastal locations along China’s coastline were investigated. For this purpose, the daily meteorological data measured at the standard 10-m height above ground for periods of 40–62 years are statistically analyzed. The East Asian Monsoon that affects almost China’s entire coastal region is considered as the leading factor determining wind energy resources. For most stations, the mean wind speed is higher in winter and lower in summer. Meanwhile, the wind direction analysis indicates that the prevalent winds in summer are southerly, while those in winter are northerly. The air densities at different coastal locations differ significantly, resulting in the difference in wind power density. The Weibull and lognormal distributions are applied to fit the yearly wind speeds. The lognormal distribution performs better than the Weibull distribution at 8 coastal stations according to two judgement criteria, the Kolmogorov–Smirnov test and absolute error (AE). Regarding the annual maximum extreme wind speed, the generalized extreme value (GEV) distribution performs better than the commonly-used Gumbel distribution. At these southeastern coastal locations, strong winds usually occur in typhoon season. These 4 coastal provinces, that is, Guangdong, Fujian, Hainan, and Zhejiang, which have abundant wind resources, are also prone to typhoon disasters.

## Introduction

During the past few decades, energy consumption increased rapidly in China with the development of economy [1, 2]. The conflict between energy demand and supply will become the major constraint on sustainable development in the future. As the nation’s most densely populated area, China's coastal regions have already faced the problem of electricity shortage [2–3]. However, China mainly relies on non-renewable fossil fuels, such as coal, petroleum, and natural gas, rather than renewable energy sources [4]. Wind energy, one of the most economical renewable forms of energy, has the potential to significantly reduce fuel costs and greenhouse gas emissions [5–7]. Wind power has already attracted widespread interest around the world [8]. China has a long coastline and thousands of nearby islands with relatively abundant wind resources in coastal regions [4, 9]. Wind power is one possible solution to respond to electricity shortage and the effects of climate change in China’s coastal regions.

Wind power density, a quantitative measure of the wind energy available for conversion to electricity by a wind turbine, is the best way to evaluate the wind energy resource [10]. Xia and Song [2] presented an overview of China’s wind energy potential and provided a map of China’s wind power density. It was founded that abundant wind resources were distributed in the southeastern coast of China [2]. A more detailed assessment of China’s wind energy resources was given in [11], where the spatial distribution of offshore wind power density was estimated using the toolkit WEST through numerical simulations. Hong and Möller [4] investigated the available offshore wind energy resources in China’s exclusive economic zone using wind speed data derived from QuikSCAT ocean wind L2B12. They also identified the shallow waters along the southern and eastern coasts of China’s mainland as suitable areas for offshore wind power in terms of wind resources and economic costs [4]. However, the above three literatures only presented the assessment of the overall wind power density in China’s coastal regions, with no other characteristics of wind energy resources mentioned. For example, typhoons frequently impact China’s southeastern coast in summer [12], and extratropical cyclones also occur in spring and autumn and affect China’s northern coast [13]. Strong winds during typhoons and extratropical cyclones have a destructive effect on wind farms. Therefore, extreme wind conditions must be taken into account during wind farm sitting [14]. In this paper, we assess both normal and extreme wind conditions for wind power to present both positive and negative aspects of wind resources in China’s coastal regions.

Wind speed and wind direction are the two basic features of the wind resource [10, 15, 16]. First, the mean wind speed and wind direction at 12 coastal locations will be statistically analyzed. The climate in China’s coastal region is the East Asian monsoon climate, which can be divided into a warm and wet summer monsoon and a cold and dry winter monsoon [17], with obvious seasonal variations. In particular, the monthly variations of the mean wind speed and wind power density will be evaluated to investigate the temporal distribution of wind power during the year. As we know, the probability distribution of wind speed is preliminarily important because it predominantly determines the performance of wind power systems [10, 16]. Once the wind speed distribution is known, the wind power potential could be easily obtained [18–21]. For this reason, a large number of studies have been published concerning the use of a variety of probability density functions (pdfs) to describe wind speed probability distributions, including the beta function, Gamma function, lognormal function, logistical function, Rayleigh function, and Weibull function [21, 22]. Among these, the Weibull distribution is the most frequently used one for modeling wind speed (typical monthly or annually) due to its two flexible parameters [21]. The Weibull shape parameter describes the width of the data distribution; the scale parameter controls the abscissa scale of the plot of data distribution [21]. Lun and Lam [23] fitted the Weibull function to wind speed data for three locations in Hong Kong from 1968 to 1997 to analyze the wind characteristics. Zhou et al. [24] applied the Weibull function to describe the hourly wind speed data from four islands in the Pearl River Delta (PRD) region of China. Oh et al. [25] presented an overall analysis of the wind energy resources in the southwest coast of the Korean Peninsula. They also chose the Weibull distribution as the probability distribution model of the wind speeds. The Weibull function was also applied in Akpınar [10] for wind power potential assessment and wind characteristic analyses for 6 coastal locations in northeastern Turkey. When the shape parameter in the Weibull distribution is kept at a constant value of 2, the Weibull distribution becomes the Rayleigh function [10, 19]. IEC suggested that the 10-min mean wind speed could be modeled by the Rayleigh function [14]. However, empirical research did not always favor the Weibull distribution [21, 22]. Luna and Church [26] found that the lognormal function was the most applicable distribution of wind speed. Garcia et al. [27] also found that the lognormal distribution was better than the Weibull distribution for wind speed data in the area of Navarre, Spain. In this study, both the Weibull and lognormal functions are applied to wind speed distribution modeling. A better distribution will be chosen according to statistical tests.

The probability distribution of extreme wind speed is the most important aspect of extreme wind conditions [14]. Extreme wind events directly influence the working order of wind turbines [28]; the probability distribution of extreme wind speed is the crucial indictor in the design of wind power plants [29]. Extreme value theory is the standard statistical technique for describing the extreme events [30]. In extreme value theory, the maximum (or minimum) values in successive periods, for example months or years, are identified and constitute the extreme events [30]. The generalized extreme value (GEV) distribution is the only possible limit distribution of such extreme values [31]. Davenport [32] was one of the earliest researchers to apply the probability and statistical theory for the determination of design wind speeds. The Gumbel distribution, which is one specific case of the GEV distribution, is most commonly adopted in structural design [33–35]. In this study, we use both the Gumbel distribution and the GEV distribution to fit the annual maximum extreme wind speeds. The likelihood ratio test will be used to compare the performances of the Gumbel and GEV distribution in fitting the annual maximum extreme wind speed. Based on the fitted distribution, the extreme wind speed of a certain recurrence period can be estimated. Finally, the types of damages of coastal wind farms caused by typhoons during the past decade are summarized. The tracks and landing locations of typhoons are also analyzed and discussed.

## Materials and Methods

### Data

**Fig 1:**
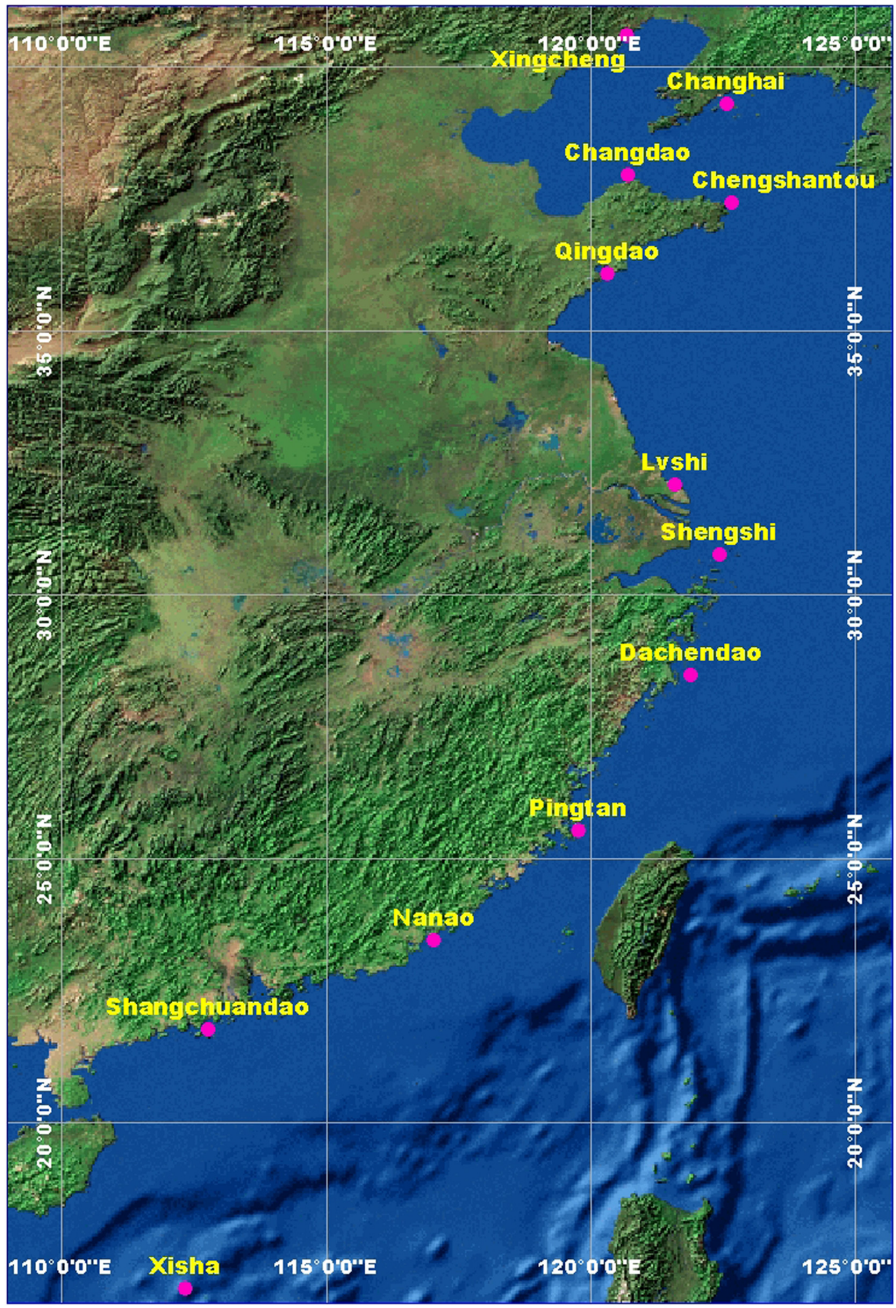
Geographic locations of all 12 coastal meteorological stations.

In this study, 12 coastal locations with meteorological stations having long-term meteorological data are selected for wind energy assessment. These meteorological stations are located either on islands or near the coastline, ranging from the Bohai Sea to the South China Sea. The geographic locations of these 12 meteorological stations are shown in Fig 1. We divide these 12 meteorological stations into two regions. Six of them are located north of the Yangtze River, belonging to the northern Region, while the other six stations are located to the south of Yangtze River, belonging to the Southern Region. Meteorological data are taken from the China Meteorological Administration (CMA) [36], and only daily records are available. All datasets are measured at a standard 10-meter height above the ground. The weather parameters of daily mean and maximum wind speed, wind direction, daily mean air temperature, mean relative humidity, and daily mean air pressure are used in this study. The detailed information regarding these 12 coastal meteorological stations is given in Table 1. The longest time series covers 62 years, and the shortest time series covers 40 years. The use of long-term measured data can effectively reduce the uncertainty of the estimation of extreme wind speed [24]. As there is no detailed information regarding the surface roughness at these coastal locations, the wind speed values at the standard height of 10 meters were not extrapolated to the hub height using the empirical power law. In addition, the records of typhoons (date, landing location, and intensity) that landed on China’s mainland during the period 1961–2013 are also collected from the CMA. Surface winds data extracted from Climate Forecast System Reanalysis (CFSR) Monthly Products (1979–2010) were also used for wind analysis at large scales. The reanalysis products were provided by the National Centers for Environmental Prediction (NCEP) [37] with spatial resolution of 2.5°*2.5°. In this study, only winds data of isobaric surface 975 mbar was used to generate the near-surface monthly wind field for surface wind analysis.

**Table 1:** Geographic coordinates and observation periods of all 12 coastal meteorological stations.

Region	Station	Longitude(E)	Latitude(N)	Altitude(m)	Observation period
Northern Region	Changhai	122°35’	39°16’	35.5	1974.01–2013.12
	Xingcheng	120°42’	40°35’	10.5	1951.01–2013.12
	Changdao	120°43’	37°56’	39.7	1961.01–2013.12
	Chengshantou	122°41’	37°24’	47.7	1952.01–2013.12
	Qingdao	120°20’	36°04’	76.0	1961.01–2013.12
	Lvshi	121°36’	32°04’	5.5	1957.01–2013.12
Southern Region	Shengshi	122°27’	30°44’	79.6	1959.01–2013.12
	Dachendao	121°54’	28°27’	86.2	1958.01–2013.12
	Pingtan	119°47’	25°31’	32.4	1954.01–2013.12
	Nanao	117°02’	23°26	7.2	1957.01–2013.12
	Shangchuandao	112°46’	21°44’	21.5	1957.11–2013.12
	Xisha	112°20’	16°50’	4.7	1952.12–2013.12

### Wind power density

The wind power density, which is proportional to the air density and the cube of the wind speed [25], can be calculated using the following equation for actual time-series data by [19, 21]:

$$P=\frac{1}{2}\rho {\bar{v}}^{3}$$ (1)

where *P* is the wind power density (units: W/m^2^); *ρ* is the air density (units: *kg*/*m* ^3^); and $\bar{v}$ is the mean wind speed (units: m/s). For actual time-series data, $\bar{v}$ is simply the average value of wind speeds:

$$\bar{v}=\frac{1}{n}\sum_{i=1}^{n}v_{i}$$ (2)

The air density is proportional to the mean air pressure (*p*, units: *Pa*) and inversely proportional to the measured absolute air temperature (*T*, units: *K*) multiplied by the gas constant for dry air (*R*_0_ = 287.058, units: *J*/*kg* ∙ *K*) [14, 25]:

$$\rho =\frac{p}{R_{0}\;T}$$ (3)

### Wind speed distribution

The probability density function (pdf) of wind speed is a function that describes the relative likelihood for wind speed to take on a given value [10]. The cumulative distribution function (cdf) of wind speed is the integral of the pdf from zero to a given wind speed value *v* [21, 22]. The probability density function and cumulative distribution function of the Weibull distribution are respectively written as [19]:

$$f\left(v\right)=\frac{k}{c}{\left(\frac{v}{c}\right)}^{k-1}\exp\left[-{\left(\frac{v}{c}\right)}^{k}\right]$$ (4)

$$F\left(v\right)=1-\exp\left[-{\left(\frac{v}{c}\right)}^{k}\right]$$ (5)

where *v* is the wind speed (units: m/s), *k* (> 0) is the dimensionless Weibull shape parameter, and *c* (> 0) is the scale parameter (units: m/s). In this paper, the traditional maximum likelihood method was adopted to estimate the two parameters *k* and *c*. All computations are implemented within the statistical computing environment R-package [38].

The lognormal distribution is a type of probability density function where the logarithm of random variable is normally distributed [21]. The probability density function *h*(*v*) and cumulative distribution function *H*(*v*) of the lognormal distribution are respectively written as [21, 22]:

$$h\left(v\right)=\frac{1}{v\sigma \sqrt{2\pi }}\exp\left\{-\frac{{\left[\ln\left(v\right)-\mu \right]}^{2}}{2\sigma^{2}}\right\}$$ (6)

$$H\left(v\right)=\frac{1}{2}+\frac{1}{2}erf\left[\frac{\ln\left(v\right)-\mu }{\sigma \sqrt{2}}\right]$$ (7)

where $\mathrm{erf}\left(x\right)=\frac{2}{\sqrt{\pi }}\int_{0}^{x}\;\exp(-t^{2})\;dt$, and the parameters *μ* and *σ* are the mean and standard deviation of the wind speed’s natural logarithm, respectively. The parameters *μ* and *σ* are also estimated using the maximum likelihood method in this study.

The goodness of fit between a theoretical probability function and observed probability distribution will be evaluated based on two judgement criteria. The Kolmogorov-Smirnov (KS) test, which is defined as the maximum error in the cumulative distribution functions [19], is firstly used

KS=max|T(v)−O(v)| (8)

where *T*(*v*) and *O*(*v*) are the probability distribution functions for wind speed not exceeding *v* in the theoretical and observed dataset, respectively. Additionally, the absolute error (AE) between *T*(*v*) and *O*(*v*) is also computed to evaluate the goodness of fitting:

$$AE=\int_{0}^{+\infty }\left|T\left(v\right)-O\left(v\right)\right|\;dv$$ (9)

### Extreme wind speed distribution

The Gumbel distribution is the commonly used extreme value distribution, which is suitable for describing the distribution of the annual maximum value [33, 39]. The probability density function of the Gumbel distribution can be written as:

$$g\left(v\right)=\frac{1}{\tau }\exp\left[-\frac{\left(v-\theta \right)}{\tau }\right]\;\exp\left\{-exp\left[-\frac{\left(v-\theta \right)}{\tau }\right]\right\}$$ (10)

The corresponding cumulative distribution function is,

$$G\left(v\right)=\exp\left\{-exp\left[-\frac{\left(v-\theta \right)}{\tau }\right]\right\}$$ (11)

where *θ* ∈ *R* is the location parameter (unit: m/s), *τ* > 0 is the scale parameter (unit: m/s), and *v* is the extreme wind speed (unit: m/s).

Jenkinson [31] combined three types of extreme value distributions into a single mathematical form producing the GEV distribution:

$$G\left(v\right)=\exp\left\{-{\left[1+\varepsilon \left(\frac{v-\theta }{\tau }\right)\right]}^{-\frac{1}{\varepsilon }}\right\}$$ (12)

The probability density function of GEV distribution is,

$$g\left(v\right)=\frac{1}{\tau }{\left[1+\varepsilon \left(\frac{v-\theta }{\tau }\right)\right]}^{-1-\frac{1}{\varepsilon }}\exp\left\{-{\left[1+\varepsilon \left(\frac{v-\theta }{\tau }\right)\right]}^{-\frac{1}{\varepsilon }}\right\}$$ (13)

for $1+\frac{\varepsilon (v-\theta )}{\tau }>0$, where *θ* ∈ *R* is also called the location parameter, *τ* > 0 is the scale parameter, and *ε* ∈ *R* is the shape parameter [30, 31, 40]. In the case of ε < 0, ε > 0 and ε = 0, Eq (12) will become the Type II extreme value distribution (Frechet distribution), Type III extreme value distribution, and Type I extreme value distribution (Gumbel distribution), respectively. In this paper, the Gumbel and GEV distributions are used to fit the annual maximum extreme wind speeds. As the Gumbel distribution is a special case of GEV distribution, the likelihood ratio test is used to compare the fitting of the two models. The null hypothesis states that the shape parameter *ε* in the GEV distribution equals to zero. Similarly, the parameters of the Gumbel and GEV distribution are also estimated using the maximum likelihood method using the statistical package R.

The return level of the extreme wind speed is defined as a value that is expected to be equaled or exceeded on average once every interval of time (*T*_0_) (with a probability of 1/*T*_0_). We call *T*_0_ the return period, and the return level can be estimated using the following equation (i.e., by inverting the GEV distribution)

$$G\left(v\right)=1-\frac{1}{T_{0}}$$ (14)

where *G*(*v*) is the cumulative distribution function of the Gumbel or GEV distribution, and *v* in Eq (14) is the return value of extreme wind speed. In IEC 61400–1 [14], extreme wind speeds are classified into three types: I, II, III. At each class, the reference extreme wind speed, which is defined as the extreme 10-min average wind speed with a recurrence period of 50 years at turbine hub height, is also proposed. In evaluating the aerodynamic load of wind turbines, two extreme wind speeds with recurrence periods of 1 year and 50 years are computed [25]. Generally, the expected lifetime of a wind turbine is approximately 20 years; therefore, the return level of extreme wind speed with a recurrence period of 20years is also estimated in this study.

## Results and Discussion

### Mean wind speed and wind power density

**Fig 2:**
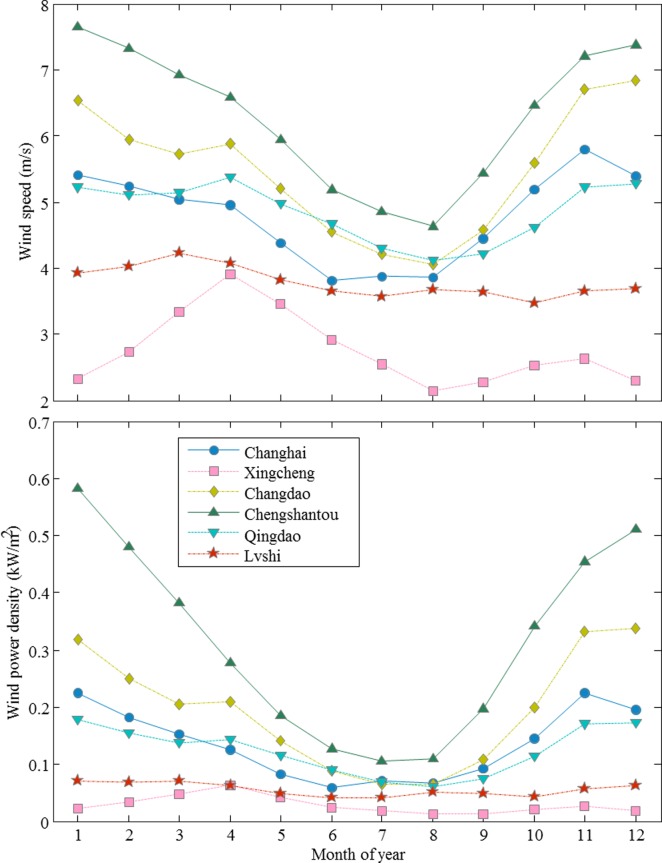
Monthly variations of the mean wind speed at a 10-m height and wind power density for the six stations Changhai, Xingcheng, Changdao, Chengshantou, Qingdao, and Lvshi in the northernern region.

The monthly variation of mean wind speed is primarily important to reveal the pattern of wind speeds during the year. In this paper, the monthly mean wind speed is calculated from the daily wind speed data according to Eq (2). The monthly variations of mean wind speed for meteorological stations in the northern region are shown in Fig 2(A). The trends of the monthly mean wind speed for Changhai, Changdao, Chengshantou, and Qingdao stations are similar. The monthly mean wind speeds are lower in summer (June, July and August) than those in other months. Chengshantou station, at the eastern end of Shandong Peninsula, has the highest wind speed in the range of 4.6 m/s to 7.8 m/s. Changdao station, located on an island in the Bohai strait, has the second highest wind speed, in the range of 4.1 m/s and 6.6 m/s. For Changhai station, located on an island in the northern Yellow Sea, the highest wind speed occurs in November (5.8 m/s); the lowest wind speed occurs in June (3.9 m/s). For Qingdao station, at the southern coast of Shandong Peninsula, the highest wind speed occurs in April (5.5 m/s) and the lowest wind speed occurs in August (4.3 m/s). In this study, wind speeds are all measured at a 10-m height above ground; therefore, and the wind speeds are also affected by local terrain. Xingcheng station is located at the northern coast of the Bohai Sea, and its northwestern side is Songling Mountains (S1 Fig). With the blocking effect of high mountains, the mean wind speed at Xingcheng station is the lowest among the 6 stations in the northern region. In March, April, and May, the mean wind speeds are approximately 3~4 m/s, but in other months, the mean wind speeds are much lower (2~3 m/s). Lvshi station, the southernmost station in north region, has the smallest variation of mean wind speed during the year (3.8~4.2 m/s). Fig 2B shows the monthly variations of wind power density calculated according to Eq (1) for these 6 stations. From Eq (1), we find that wind speed contributes more than the air density to wind power; therefore, the monthly variations of wind power density are almost identical to that of mean wind speeds. Chengshantou station has much more wind energy than the other stations, reaching up to 0.59 *kW*/*m* ^2^ in January. For Xingcheng station, as the wind speed is the lowest, the wind power density is also the lowest among the 6 stations. Lvshi station is located at the southern Jiangsu coast, the estimated wind power density at a 10-m height above ground in this study is 0.06~0.1 *kW*/*m* ^2^. The altitude is only 5.5 m above sea level, and the surface roughness is low. It was reported that the wind power density could reach 0.25 *kW*/*m* ^2^ at the height of 40 m along the southern Jiangsu coast [11]. The influence of air density on wind power density can also be observed in Fig 2. For example, although the mean wind speed in Changhai station is lower than that in Qingdao station in April, the wind power density is higher due to the higher air density. In addition, the standard deviations of the monthly mean wind speeds and wind power densities are given in S1 Table. A larger monthly mean wind speed or wind power density has a larger standard deviation.

**Fig 3:**
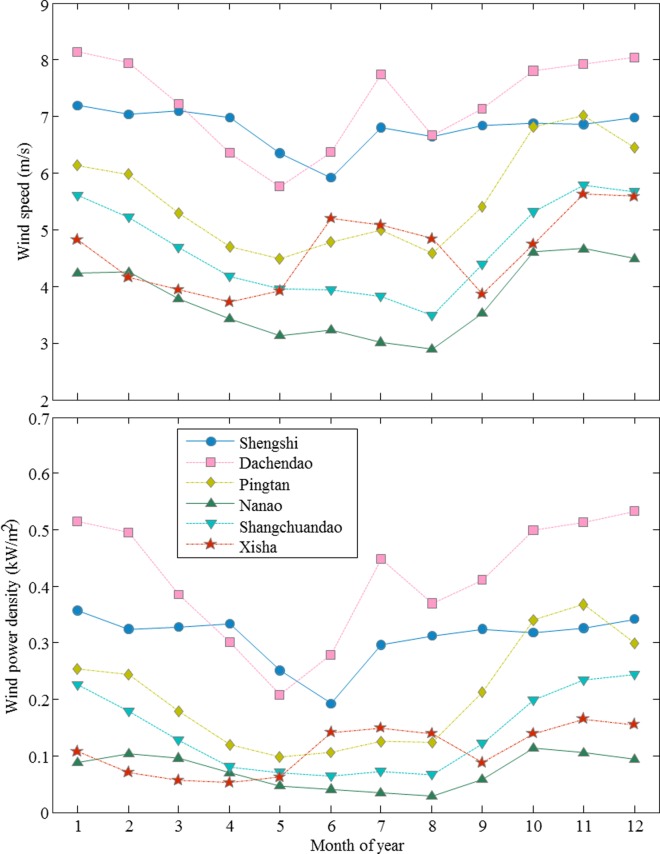
Monthly variations of mean wind speed at a 10-m height and wind power density for the six stations Shengshi, Dachendao, Pingtan, Nanao, Shangchuandao, and Xisha in the southern region.

Fig 3 shows the monthly variations of the mean wind speed and wind power density for the 6 stations in the southern region. Analogously, the standard deviations of the monthly mean wind speeds and wind power densities for these 6 stations are also given in S1 Table. The trends for the monthly mean wind speed for Pingtan, Nanao, and Shangchundao stations are similar. These 3 stations are located near the coast of China’s mainland from the Taiwan Strait to the Pearl River Delta and share similar climate. The mean wind speeds are lower in May, June, July, and August, but higher in October, November, December, January, and February. The maximum monthly mean wind speed occurs in November. Zhou et al. [24] conclude that the cooling effect of the Asiatic continent produces a higher wind speed in winter and that its heating effect results in a relatively low wind speed in summer. Shengshi station and Dachendao station are both located on islands; therefore, most of the monthly mean wind speed values are over 6m/s. For Dachendao station, the monthly mean wind speed first decreases from January to May, and then increases from June to July as the summer monsoon strengthens. When the summer monsoon becomes weaker in August, the monthly mean wind speed increases again from August to December. For Shengshi station, the maximum monthly mean wind speed is 7.1 m/s, while the minimum monthly mean wind speed is 6 m/s. Xisha station is far away from the mainland, and the monthly mean speed exhibits an obvious monsoon feature. There are two peaks in the monthly mean wind speeds corresponding to 5.3 m/s in June and 5.6 m/s in November. The monthly variations of wind power density at these 6 stations, plotted in Fig 3(B), are similar to the variations of mean wind speed, shown in Fig 3(A). In March and April, although the mean wind speed in Xisha station is higher than that in Nanao station, the wind power density is lower due to the warmer and wetter weather conditions.

The climate of China is mainly influenced by East Asian monsoon, which can be divided into a warm and wet summer monsoon and a cold and dry winter monsoon [41]. The temperature difference between the Asian continent and the Pacific Ocean is considered as the driving forces of the East Asian monsoon. In S2 Fig, we show the near-surface monthly wind field of the East Asian region (100°~140°E, 10°~60° N). In winter, the winds are stronger and almost northerly. But in summer, the winds are weaker and almost southeasterly. These characteristics are consistent with the patterns of the monthly variation of mean wind speed computed using the meteorological data at the 12 meteorological stations. It is concluded that the East Asian Monsoon is the major factor that determines the annual pattern of wind energy resource in China’s coastal region.

As we know, it is also necessary to assess the diurnal variations of wind speeds in addition to the monthly or seasonal variations for small isolated power systems [10, 20]. Due to the lack of hourly or 10-min wind speed data, the diurnal variations of mean wind speeds and wind power density at these coastal locations are not presented in this study. Moreover, in a practical assessment of the wind potential at a specific site, the surface roughness should also be surveyed to determine the optimal the hub height of wind turbines and maximize the economic feasibility because wind speed varies by height depending on the surface roughness [25]. The vertical wind profile is usually described by a power law [10, 25]; therefore, the available wind speeds at standard height are adjusted to the wind turbine hub height accordingly. In this paper, the terrain of the selected 12 stations is different and the information regarding the surface roughness is insufficient. Finally, China has a long coastline extending from the temperate zone to the tropical zone; the climate at different latitudes varies significantly. As a result, the air density varies under different climatic conditions. Therefore, air density should also be seriously considered in the wind power potential assessment at different coastal locations.

### Wind direction

**Fig 4:**
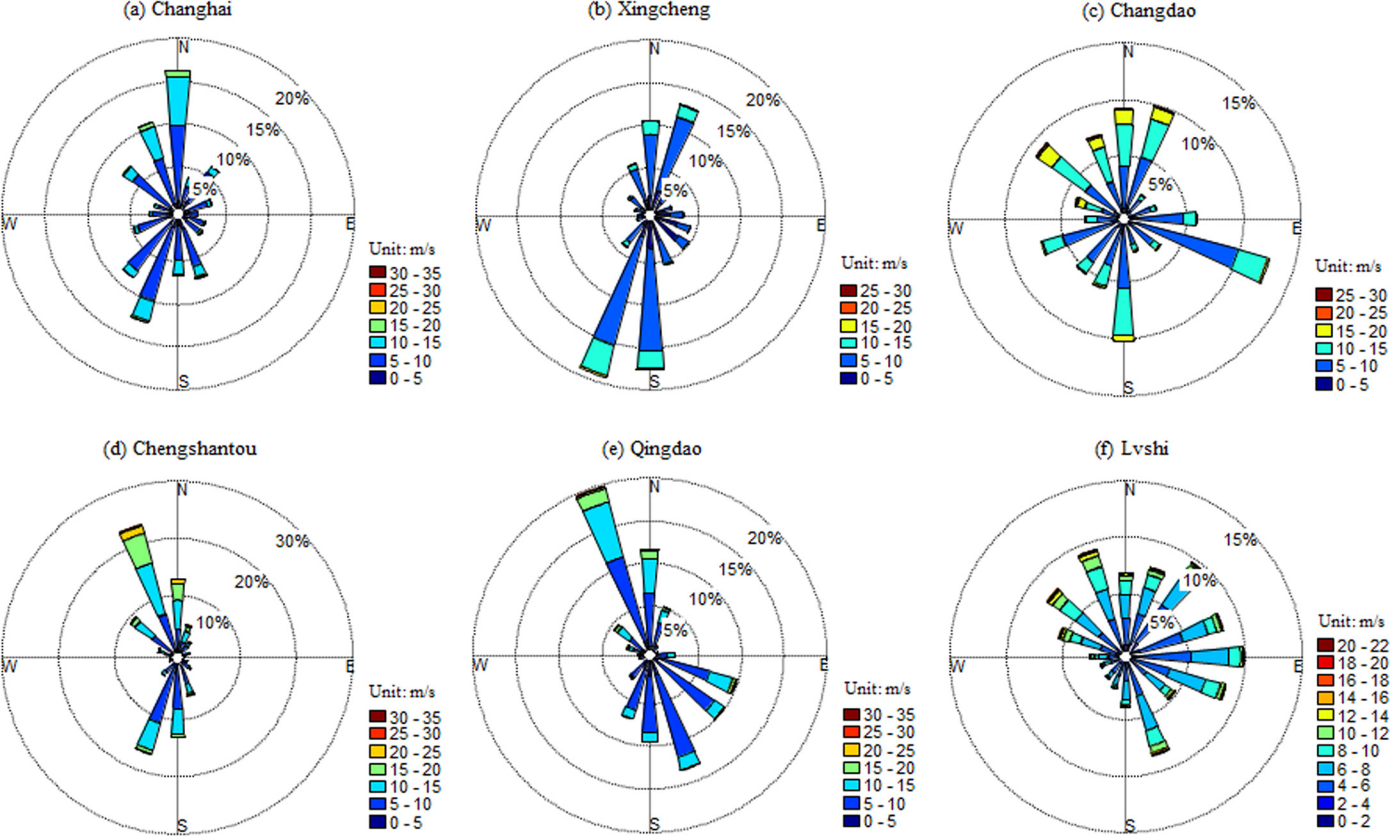
Wind rose diagrams for the six stations Changhai, Xingcheng, Changdao, Chengshantou, Qingdao, and Lvshi in the northern region.

**Fig 5:**
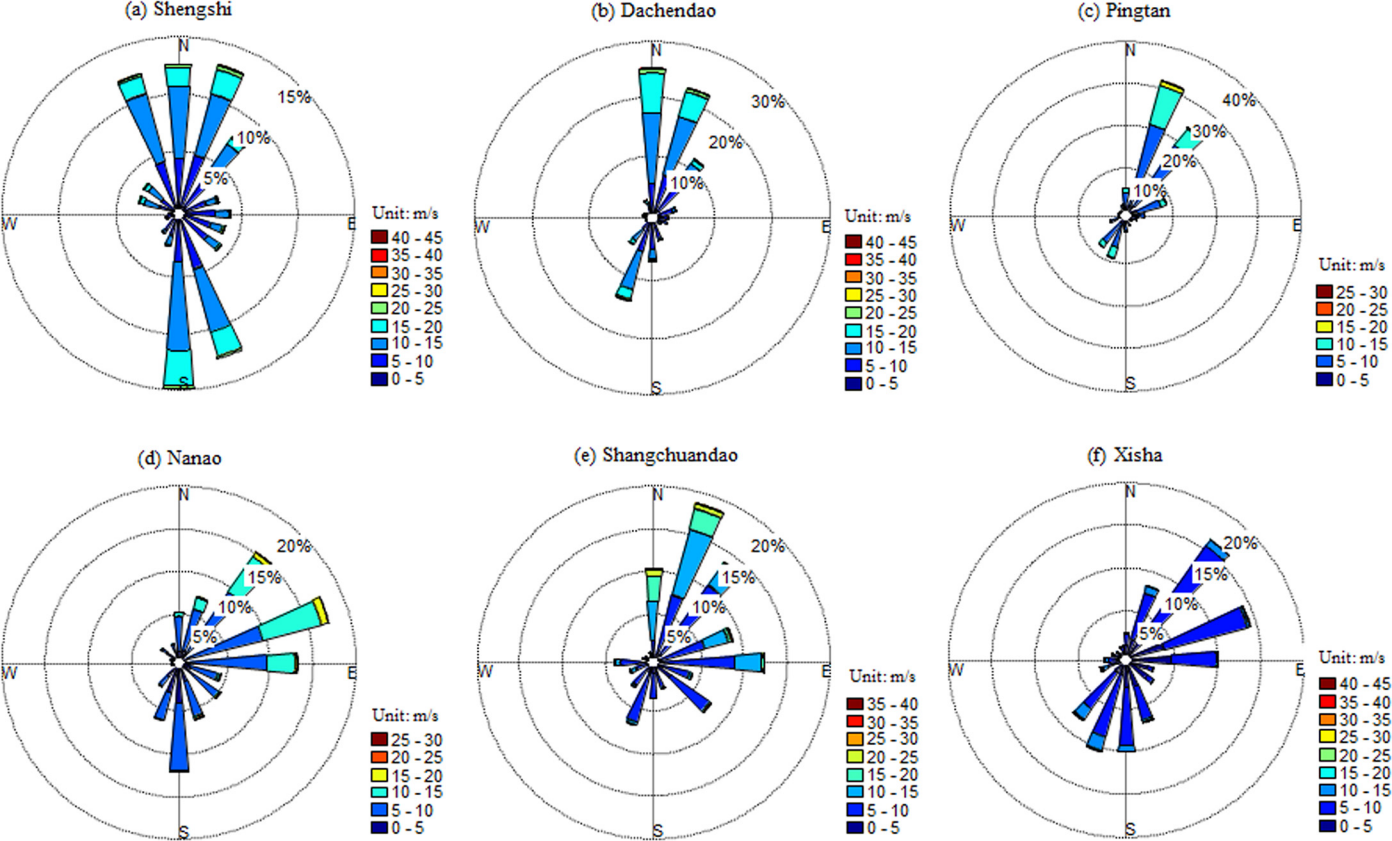
Wind rose diagrams for the six stations Shengshi, Dachendao, Pingtan, Nanao, Shangchuandao, and Xisha in the southern region.

To evaluate wind energy properly, it is of great importance to investigate the wind direction with other parameters [20]. A wind rose is a diagram showing the temporal distribution of wind direction and the azimuthal distribution of wind speed at a given location [16]. Similar to wind speed, wind roses also vary with location and are known as a form of meteorological fingerprint [10]. Examining the wind rose as well as understanding its message correctly is important for setting up wind turbines. If a site has a dominant direction then wind turbines should be aligned against this direction. To construct wind roses, all data of the time series of wind speed and wind direction are used. The most common form of the wind rose diagram consists of 16 equally spaced sectors, each of which covers an arc of 22.5°. The largest sectors identify the prevailing wind direction [16]. Figs 4 and 5 show the wind rose diagrams for these 12 stations in the northern and southern regions, respectively. In the northern region, winds blow predominately from the north (N), south-southwest (SSW), north-southwest (NSW), and north-southwest (NSW), for Changhai, Xingcheng. Chengshantou, and Qingdao stations, respectively. The wind directions at these four stations represent the typical features of the East Asian monsoon climate (S2 Fig). For Changdao station, the wind direction in different season varies obviously. The prevailing wind directions are south (S), east-southeast (ESE), and northwest (NW) in April to May, June to July, and November to January, respectively. Chaodao station is located on a coastal island with a varied topography that might affect wind directions (S3 Fig). But the wind directions are relatively concentrated on the mountain ridge; therefore, wind power plants have already been constructed and put into operation (S3 Fig). For Lvshi station, there is no prevailing wind direction yet. Lvshi station is close to the moth of Yangtze River, and the topography is very flat (S4 Fig). Yu et al. [42] assessed the wind energy resource on Chongming Island, which is about 40km away from Lvshi station, using wind data measured at 50-m meteorological mast. They got similar wind rose map without prevailing wind direction. Additionally, we also presented the wind rose maps of another two near stations, Nantong and Baoshan (S4 Fig), with shorter time series of daily weather parameters (1991–2013). No prevailing wind directions are observed either. However, the reason why there was no prevailing wind direction at this region was not clear. In the southern region, the imprint of the East Asian monsoon on wind direction is also very obvious (S2 Fig). For Shengshi station, NNW, N, NNE, SSE and S account for more than 60% of the windward directions. For Dachendao station, N, NNE, and SSW account for more than 65% of the windward directions. For Pingtan and Shangchuandao stations, the probable wind directions are NNE and NE, respectively. For Nanao and Xisha stations, the prevailing wind directions are ENE and NE, respectively. Generally, the prevalent winds in summer are southerly, and those in winter are northerly for most stations in the northern and southern regions.

### Wind speed distribution

**Fig 6:**
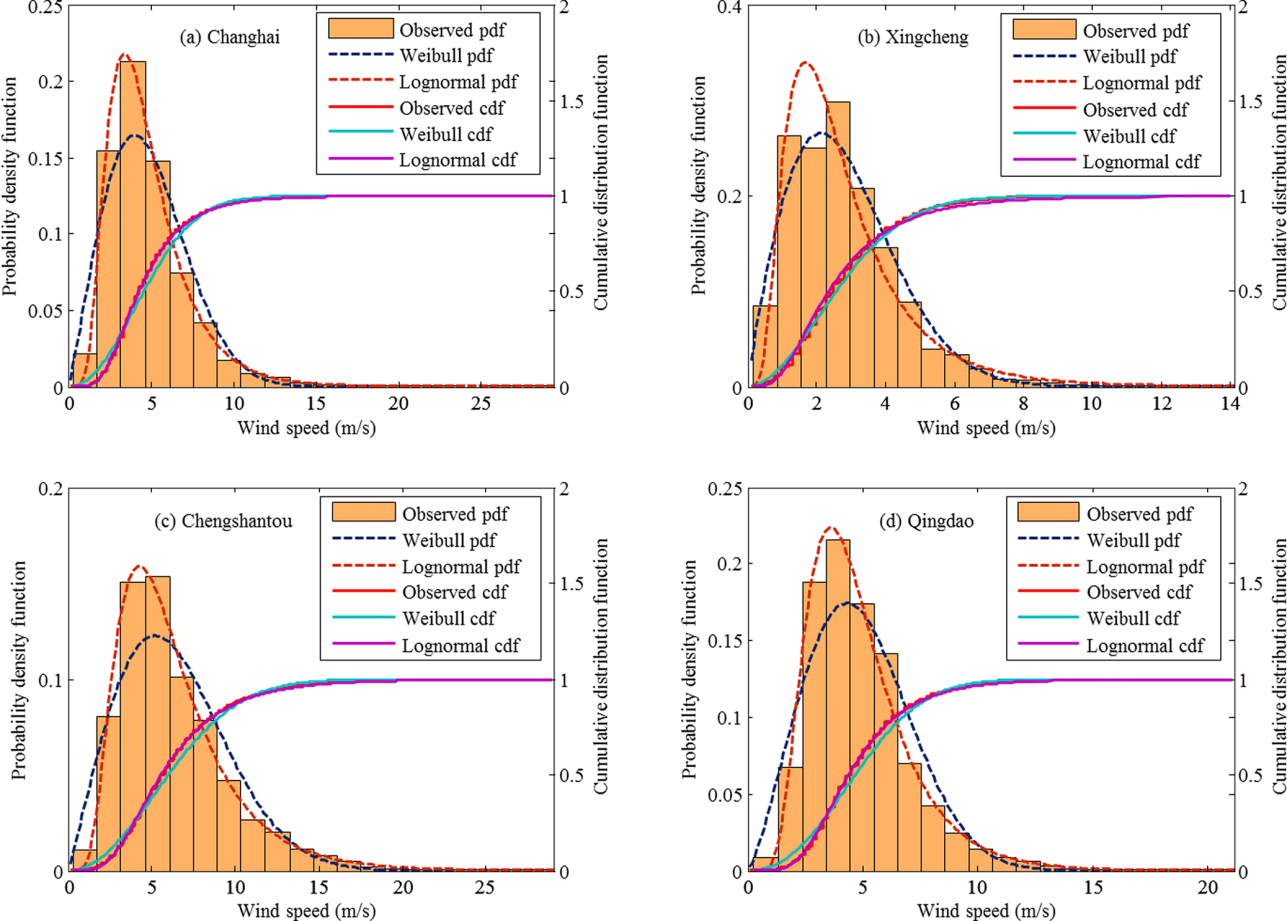
Observed probability distribution of the yearly wind speeds vs. fitted probability density functions (pdfs) and cumulative distribution functions (cdfs) of the Weibull distribution and the lognormal distribution for stations of Changhai, Xingcheng, Chengshantou, and Lvshi in the northern region.

**Fig 7:**
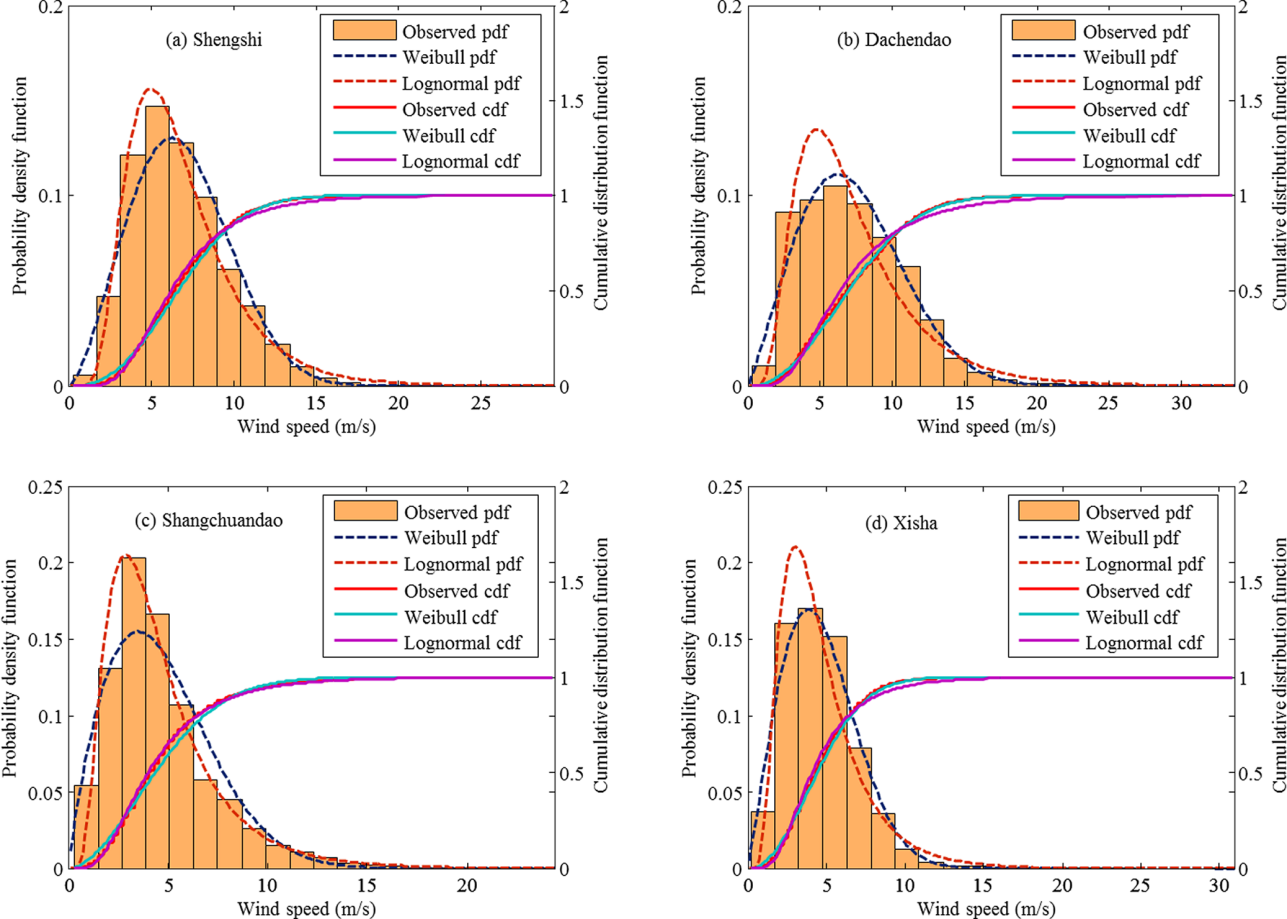
Observed probability distribution of the yearly wind speeds vs. fitted probability density functions (pdfs) and cumulative distribution functions (cdfs) of the Weibull distribution and the lognormal distribution for stations of Shengshi, Dachendao, Shangchuandao, and Xisha in the southern region.

Two probability distributions, Weibull and lognormal, are applied to fit the yearly wind speed data. The estimates of the model parameters and the corresponding criteria KS and AE for each fitting are listed in Table 2. In the present study, the judgement criterion KS or AE indicate how a theoretical probability density function matches with the observed wind speed distribution, where a smaller KS or AE indicates a better fitting. In the northern region, the lognormal distribution performs better than the Weibull distribution in fitting yearly wind speeds for most stations, except for Xingcheng station. Fig 6 compares the theoretical probability density functions with the observed wind speed histograms of yearly wind speeds for 4 stations in the northern region. Therein, the corresponding cumulative distribution functions are also plotted for comparison. In Fig 6(B), the Weibull distribution is observed to be consistent with observed distribution of wind speeds for Xingcheng station. In Fig 6A, 6C and 6D, the lognormal distribution is closer to the observed distribution than the Weibull distribution. In the southern region, we find that the Weibull distribution performs better in fitting yearly wind speeds for Shengshi, Dachendao, and Xisha stations; while for the other 3 stations, the lognormal distribution performs better in terms of the criteria KS and AE. Fig 7 shows the fitted probability density (and cumulative distribution) functions and the observed probability distribution of yearly wind speeds for 4 stations in the southern region.

**Table 2:** Estimate of parameters in Weibull distribution and lognormal distribution by fitting yearly mean wind speeds for all 12 coastal stations and the results of Kolmogorov-Smirnov (KS) test and absolute error (AE) criterion.

Station	Weibull model				Lognormal model			
	*c*	*k*	KS	AE	*μ*	*σ*	KS	AE
Changhai	5.4163	2.1197	0.0814	0.3418	1.4516	0.4816	**0.0360**	**0.079**
Xingcheng	3.1332	1.9079	**0.0637**	**0.1311**	0.8601	0.592	0.0695	0.166
Changdao	6.2164	2.1208	0.0642	0.3085	1.5815	0.5061	**0.0422**	**0.1598**
Chengshantou	7.1395	2.064	0.067	0.3949	1.7138	0.5169	**0.0390**	**0.1582**
Qingdao	5.4875	2.333	0.0783	0.313	1.4836	0.4454	**0.0378**	**0.089**
Lvshi	4.2805	2.4144	0.0774	0.1957	1.237	0.4501	**0.0591**	**0.1221**
Shengshi	7.6818	2.4751	**0.0434**	**0.1953**	1.8203	0.4604	0.0524	0.3125
Dachendao	8.2196	2.1925	**0.0412**	**0.1687**	1.8522	0.5371	0.0726	0.5703
Pingtan	6.2933	2.0223	0.0528	0.2452	1.5743	0.5506	**0.0473**	**0.2417**
Nanao	4.2659	1.8345	0.0679	0.1912	1.158	0.607	**0.0444**	**0.1828**
Shangchuandao	5.2826	1.8599	0.0699	0.3086	1.3841	0.5772	**0.0497**	**0.1583**
Xisha	5.2425	2.1065	**0.0439**	**0.1527**	1.4023	0.5394	0.063	0.2912

For the total 12 stations, the lognormal distribution performs better than the Weibull distribution at 8 stations. The goodness of fitting of different probability distributions in fitting observed wind speeds is mainly determined the actual frequency distribution of wind speeds. Actually, the frequency distribution of wind speeds is also an important characteristic of wind energy resource. In this study, we use the maximum and absolute error between the observed and fitted cumulative distribution functions to evaluate the goodness of fitting. When a different evaluation criterion is used, the selected optimal distribution for wind speeds usually changes [22]. In practice, in addition to the quantitative evaluation criteria, the visual inspection is also very important in selecting a better probability distribution of wind speeds. However, the difference between different cumulative probability distributions cannot be easily detected by visual inspection. Therefore, the difference between probability density function and observed frequency distribution is usually preferred for visual inspection. The observed frequency distribution of wind speed is demonstrated using histograms, and its shape varies with the number of bins. Therefore, the result of visual inspection on probability density functions and frequency distribution is not always consistent with that of the statistical test such as KS test. An example is Fig 7(A), where the Weibull distribution does not fit the observed wind speeds satisfactorily as shown in terms of the criteria KS and AE in Table 2. In practical wind energy assessment, there is no generally-accepted probability distribution that is suitable for all wind speed data. IEC [14] recommended using the simplest probability distribution, Rayleigh distribution, to fit 10-min mean wind speed. Rayleigh distribution is a special case of the Weibull distribution with only one parameter; therefore, it is easily to implement model fitting and parameter estimation. With the development of statistical software, fitting a probability distribution function to a long time series of wind speeds and estimating the model parameters is not a difficult task any more. The free statistical software, R-package, can quickly implement model fitting.

### Extreme wind speed

**Fig 8:**
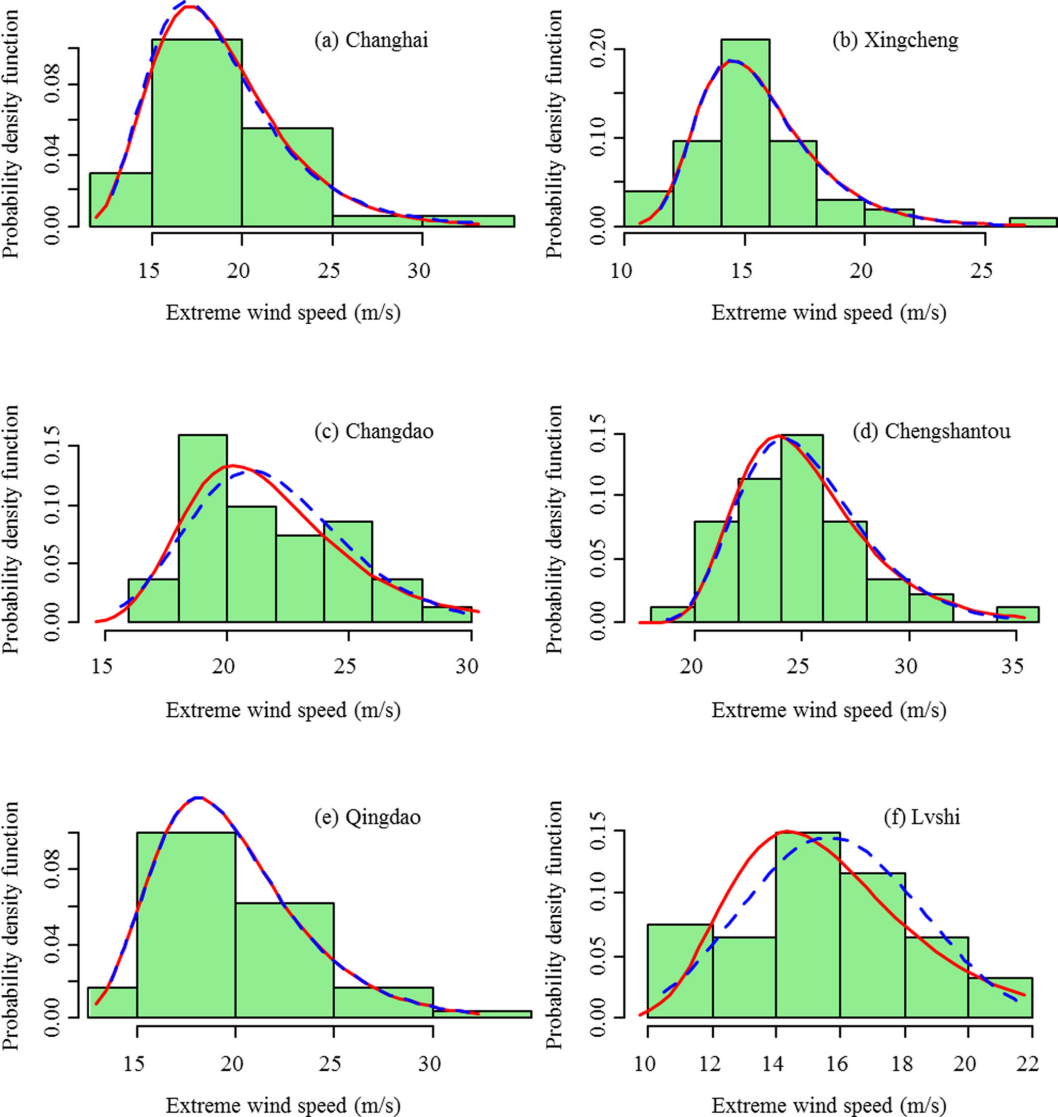
Observed probability distribution of the annual maximum extreme wind speeds vs. fitted probability density functions (pdfs) of the Gumbel distribution (solid lines) and the GEV distribution (dashed lines) for the stations of Changhai, Xingcheng, Chengshantou, and Lvshi in the northern region.

**Fig 9:**
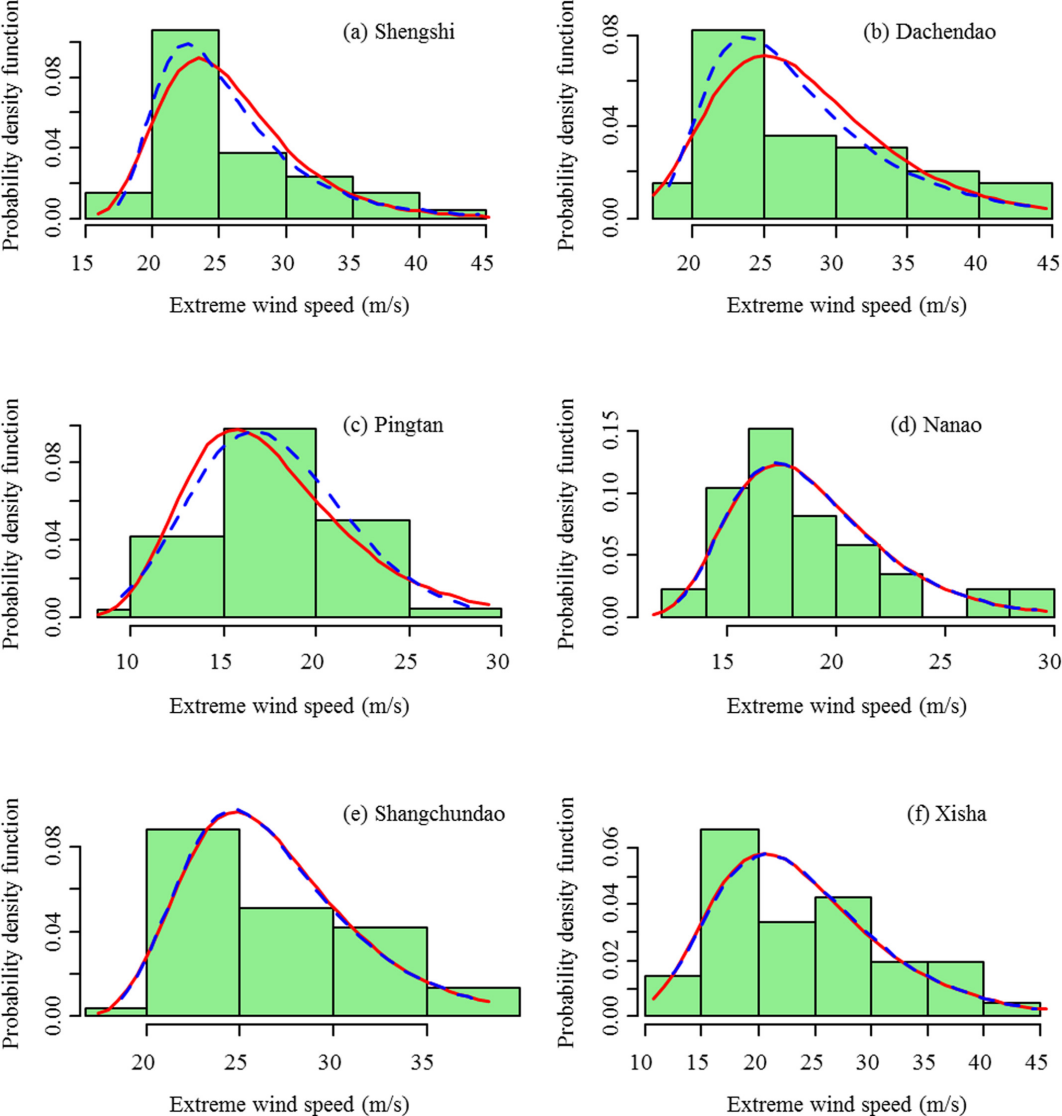
Observed probability distribution of annual maximum extreme wind speeds vs. fitted probability density functions (pdfs) of the Gumbel distribution (solid lines) and the GEV distribution (dashed lines) for the stations of Shengshi, Dachendao, Pingtan, Nanao, Shangchuandao, and Xisha in the southern region.

The annual maximum extreme wind speeds at a 10-m height are fitted to the Gumbel distribution and the GEV distribution. The estimated values of parameters are listed in Table 3. Figs 8 and 9 compare the probability density distributions of the Gumbel distribution and the GEV distribution with the empirical distribution for stations in the northern region and southern region, respectively. From Figs 8 and 9, the Gumbel distribution and GEV distribution are almost the same for the Changhai, Xingcheng, Chengshantou, Qingdao, Shengshi, Dachendao, Nanao, and Shangchuandao stations. The estimate of the shape parameter *ε* also verifies this result (Table 3). The likelihood ratio test further reveals that the differences between the fitted Gumbel distribution and the GEV distribution are not significant, except for the Lvshi station (*p* < 0.05, Table 3). The Gumbel distribution and GEV distribution are nested probability distributions, where the Gumbel is the simpler one with fewer parameters. In most cases, the Gumbel distribution is capable of describing the distribution of the extreme wind speed; however, we find that the GEV distribution model is better than the Gumbel distribution in this study. Using the statistical package R, the fitting of the empirical data to GEV distribution is not as complicated as fitting to the Gumbel distribution. We recommend using the GEV distribution for extreme value analysis.

**Table 3:** Estimates of the parameters in the Gumbel distribution and the generalized extreme value (GEV) distribution by fitting the annual maximum extreme wind speeds for all 12 coastal stations and the results of the likelihood ratio test.

Station	Gumbel model		GEV model			Likelihood ration test	
	*θ*	*τ*	*θ*	*τ*	*ε*	p-value
Changhai	17.039	2.957	16.964	2.905	0.046	0.706
Xingcheng	14.499	1.962	14.478	1.951	0.019	0.827
Changdao	20.276	2.757	20.494	2.881	-0.145	0.306
Chengshantou	23.811	2.489	23.899	2.528	-0.065	0.532
Qingdao	18.078	3.102	18.073	3.098	0.003	0.984
Lvshi	14.37	2.464	14.753	2.641	-0.281	**0.034**
Shengshi	23.427	4.034	23.09	3.744	0.163	0.173
Dachendao	24.989	5.175	24.495	4.726	0.187	0.279
Pingtan	15.618	3.754	15.942	3.851	-0.161	0.145
Nanao	17.312	2.975	17.284	2.959	0.018	0.873
Shangchuandao	24.741	3.806	24.708	3.78	0.017	0.918
Xisha	20.525	6.326	20.566	6.359	-0.012	0.94

**Fig 10:**
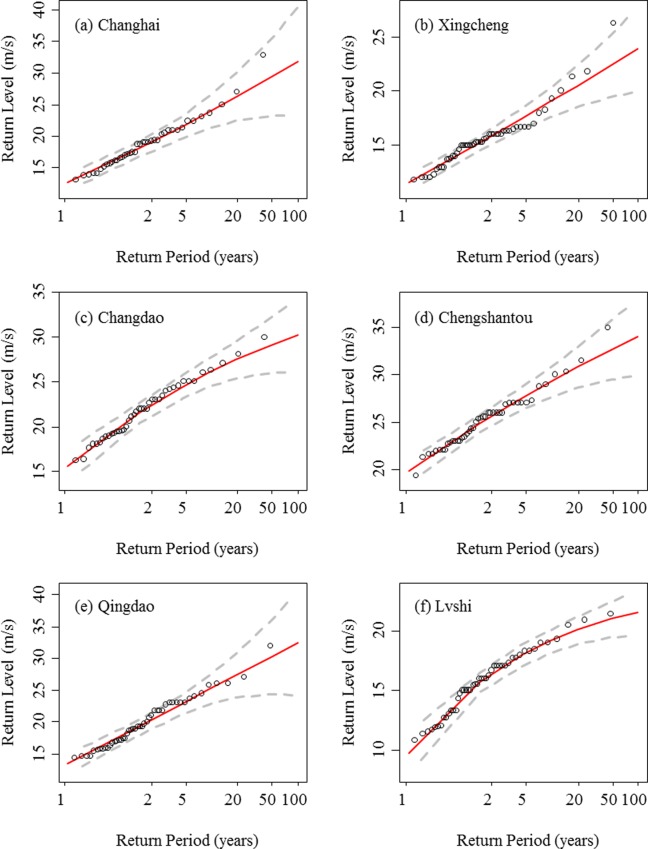
Return levels of the extreme wind speed predicted by the GEV distribution (solid lines) for the stations of Changhai, Xingcheng, Chengshantou, and Lvshi in the northern region. The circles indicate the observed annual maximum extreme wind speeds, and the dashed lines indicate the 95% confidence intervals of the GEV prediction.

**Fig 11:**
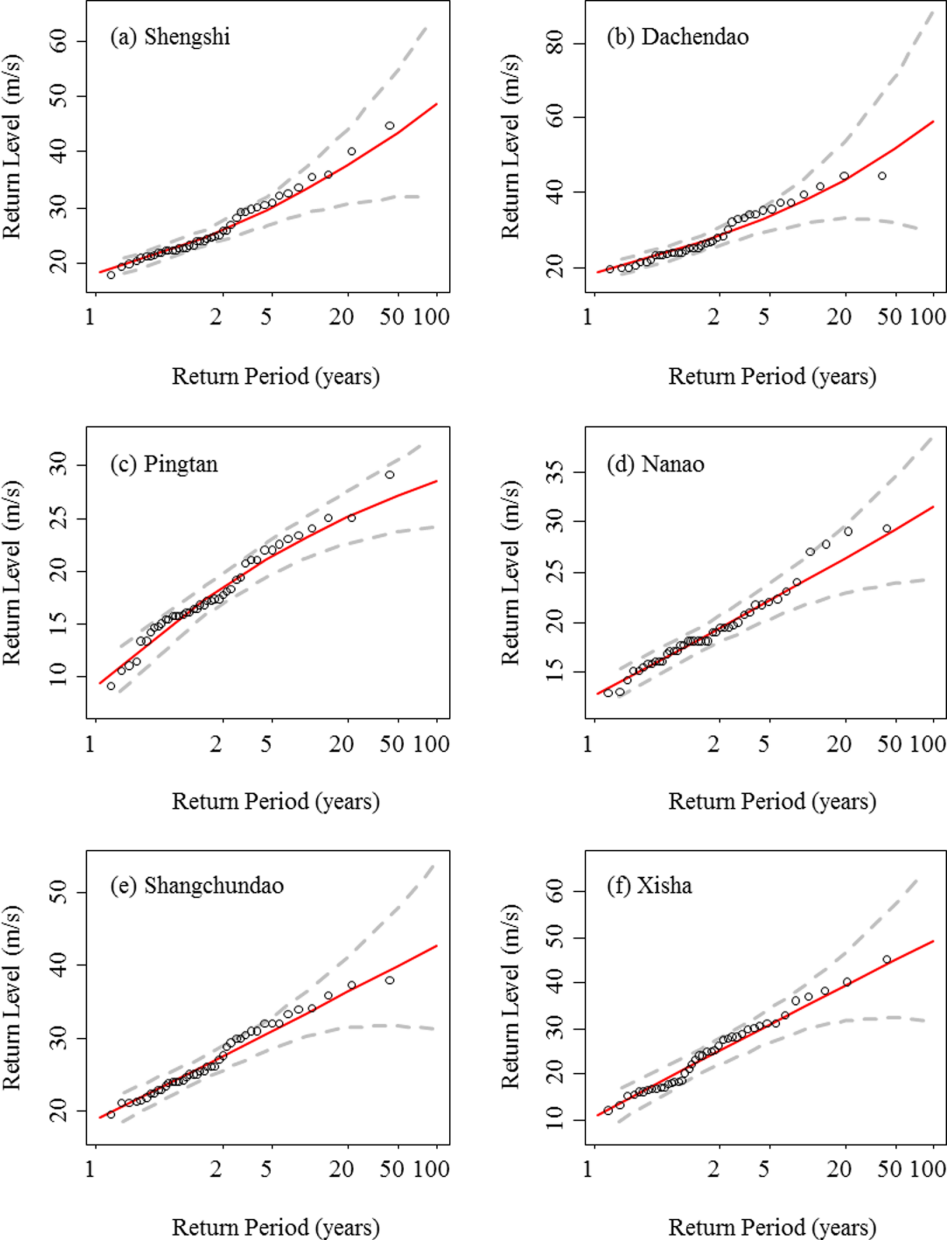
Return levels of extreme wind speed predicted by the GEV distribution (solid lines) for the stations of Shengshi, Dachendao, Pingtan, Nanao, Shangchuandao, and Xisha in the southern region. The circles indicate the observed annual maximum extreme wind speeds, and the dashed lines indicate the 95% confidence intervals of the GEV prediction.

The return levels of extreme wind speed for the northern region and southern region are presented in Figs 10 and 11, respectively. Table 4 shows the return level of extreme wind speed at a 10-m height above ground for 1-year, 20-year, and 50-year periods. In the northern region, the predicted highest 1-year, 20-year, and 50-year return levels of extreme wind speeds are 19.83, 30.72, and 32.61 m/s, respectively, at Chengshantou station. The recorded maximum extreme wind speed was 34.9 m/s on August 28^th^, 2008, when typhoon “Bolaven” passed Shandong peninsula. The lowest 1-year, 20-year, and 50-year return level of extreme wind speed are 9.71, 20.07, and 21.00 m/s at Lvshi station. In the southern region, the lowest 1-year, 20-year, and 50-year return levels of extreme wind speeds are 9.27, 25.04, and 27.11m/s, respectively, at Pingtan station. However, the highest return levels of extreme wind speed for different return periods are not consistent. The highest 1-year return level of extreme wind speed is 18.99 m/s at Shangchuandao station. The highest 20-year and 50-year return levels of extreme wind speeds are 43.26 and 51.63 m/s at Dachendao station. The recorded maximum extreme wind speed was 44.1 m/s on Aug. 12^th^, 2004 and Sept. 11^th^, 2005 corresponding to typhoons “Rananim” and “Khanun”, respectively. Although the observed maximum extreme wind speeds are not exactly equal to the predicted extreme wind speeds, they almost fall within the 95% confidence interval of the GEV prediction (Figs 10 and 11).

**Table 4:** Return levels of the extreme wind speed predicted by the GEV distribution for at all 12 coastal stations.

Station	Return level of extreme wind speed (m/s)		
	1-year	20-year	50-year
Changhai	12.68	26.21	29.39
Xingcheng	11.54	20.43	22.38
Changdao	15.56	27.45	29.09
Chengshantou	**19.83**	**30.72**	**32.61**
Qingdao	13.35	27.31	30.23
Lvshi	9.71	20.07	21.00
Shengshi	18.02	37.39	43.50
Dachendao	18.21	**43.26**	**51.63**
Pingtan	9.27	25.04	27.11
Nanao	12.82	26.31	29.24
Shangchuandao	**18.99**	36.22	39.95
Xisha	10.75	39.11	44.79

### Landfalling typhoons

**Fig 12:**
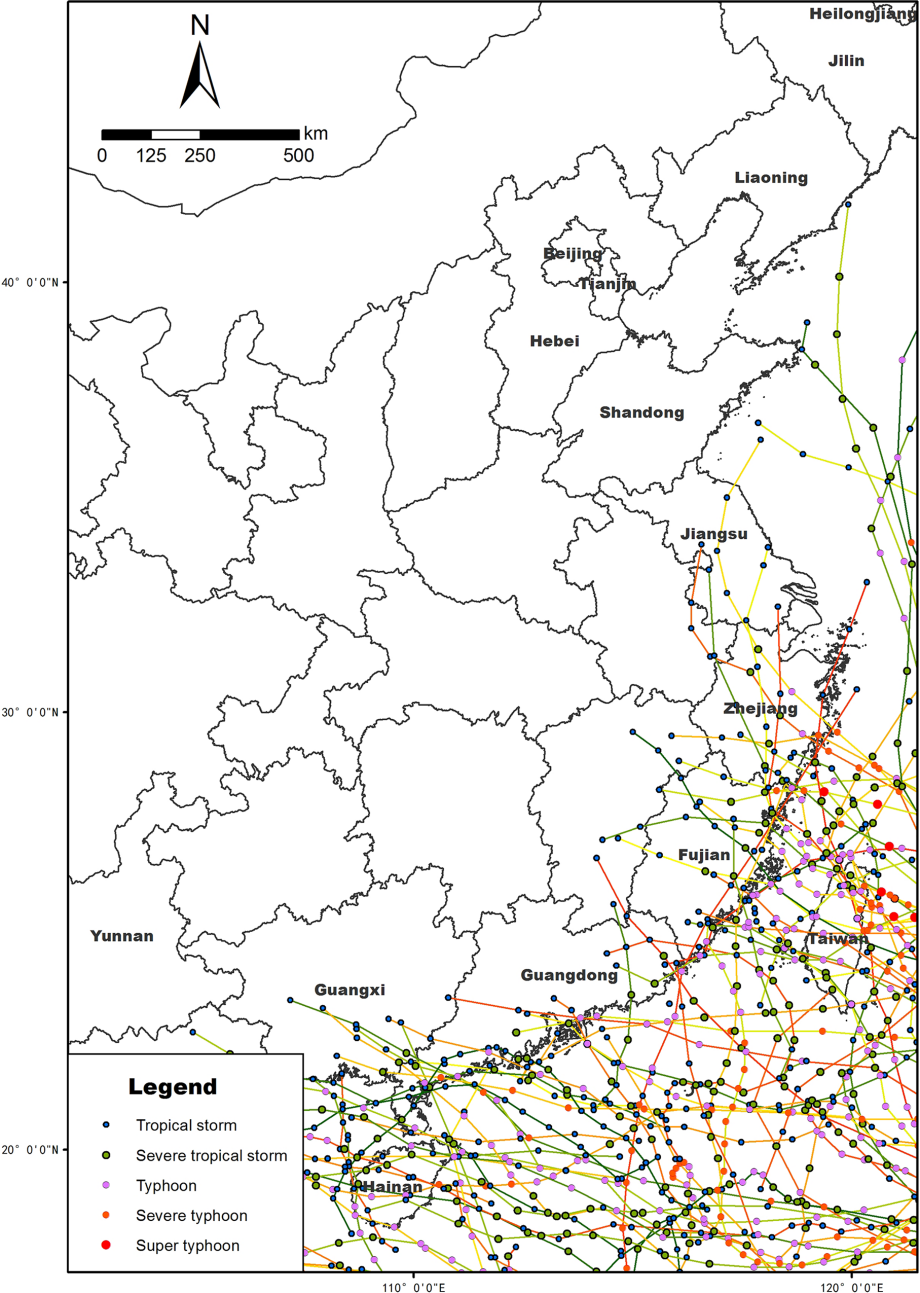
Tracks of all of the tropical storms (TSs), severe tropical storms (STSs), typhoons (TYs), severe typhoons (STYs), and super typhoons (Super TYs) in China’s coastal regions from 2002 to 2011.

A typhoon is a mature tropical cyclone that develops in the western part of the North Pacific Ocean. According to the intensity (wind speed), a typhoon can be divided into 6 classifications [22, 43, 44]: tropical depression (TD), tropical storm (TS), severe tropical storm (STS), typhoon (TY), severe typhoon (STY), and super typhoon (Super TY). The main effects of typhoon include heavy rain and strong wind causing death and destruction. Currently, increasing number of wind farms are established in tropical cyclone zones, causing the wind turbine and its support structure to be under the threat of typhoon. The destructive effects of typhoons on coastal wind farms include wind turbine failure, blade fracture, and tower collapse. Massive losses in coastal wind farms caused by typhoon have been occasionally reported in recent years in China. Table 5 summarizes the publicly reported damage of wind turbines in south-east China from 2003 to 2014. On Aug. 10^th^, 2006, all 28 wind turbines were destroyed when super typhoon “Saomei” passed the Heidingshan wind farms in Zhejiang province. On July 18^th^, 2014, super typhoon “Rammasun” landed in Hainan province and Guangdong province blowing down 14 towers. Table 6 summarizes the landing locations and intensity of typhoons (STS, TY, STY and Super TY) in the period 1961 to 2013. The landing locations of most typhoons are found to be distributed in four southeastern provinces: Guangdong, Fujian, Hainan, and Zhejiang. To illustrate typhoon landing locations clearly, we show the tracks of TD, STS, TY, STY, and Super TY in the coastal regions from 2002 to 2011in Fig 12. Note that many typhoons also impact Guangxi or other inland provinces after landing on Guangdong.

**Table 5:** Damage of the wind turbines in coastal wind farms caused by typhoons in recent years. The symbol “-” represents no data available.

Landing date	Typhoon	Wind Farm	Number of turbine failure	Number of blade fracture	Number of tower collapse
2003.9.3	Dujuan	Hongshuwan	4	9	0
2006.8.10	Saomei	Hedingshan	13	10	5
2010.10.23	Meji	Liuao	4	1	1
2013.9.22	Usagi	Hongshuwan	-	9	8
2014.7.18	Rammasun	Wenchang	-	3	1
2014.7.18	Rammasun	Xunwen	-	5	13

**Table 6:** Summary of landing locations of typhoons (STS, TY, STY and Super TY) in the period of 1961–2013. The number in the table is the number of times that typhoons landed on the province. Abbreviations: super typhoon (Super TY), severe typhoon (STY), typhoon (TY), and severe tropical storm (STD).

Province	Super TY	STY	TY	STS	Total
Hainan	3	8	28	24	63
Guangxi	0	0	0	1	1
Guangdong	3	11	53	60	127
Fujian	4	9	31	23	67
Zhejiang	1	8	11	10	30
Shanghai	0	0	1	1	2
Jiangsu	0	0	2	1	3
Shandong	0	0	0	6	6
Liaoning	0	0	0	1	1

Because the destructive effects of typhoons on coastal wind farms, typhoons should be carefully considered during wind farm sitting and structural design. Numerical climate models have predicted that ongoing global warming could change the intensity, frequency and prevailing tracks of typhoons [45–49]. Chan et al. [48] found that there was a positive and statistically significant trend of the annual number of typhoons those landing on the East China coast during this period 1450–1949. Periodical oscillations on centennial to decadal timescales were also observed. Wu et al. [49] found that the two prevailing typhoon tracks in the western North Pacific have shifted westward significantly and more typhoons would influence the subtropical East Asia. As shown in previous section, extreme wind speeds are usually caused by typhoons in China’s coastal zone. When GEV distribution based on the extreme values theory is used for return level estimation, there was a basic assumption of stationarity [50]. Because of the significant impact of global warming on typhoons, the time series of annual maximum extreme wind speeds will become nonstationary. Thus, more sophisticated statistical methods are preferred for modeling nonstationary extremes during engineering design of wind farms in China’s coastal zone.

## Conclusions

This study investigated the wind conditions at 12 coastal locations along China’s coastline. These coastal locations are divided into the northern region and southern region, and long-term (40 to 62 years) meteorological data at the standard 10-m height above ground were used for statistical analysis. The monthly variations of mean wind speed and wind power density were first investigated. Later, wind directions were analyzed. Next, two probability distributions, Weibull and lognormal, were used to fit the yearly mean wind speeds to study the wind speed frequency distribution. Finally, extreme wind conditions were also elucidated, and the destructive effect of typhoons on coastal wind farms was also discussed. The crucial outcomes of this study are summarized as follows:

- Based on the monthly mean wind speed, we find that the mean wind speed is higher in winter but lower in summer for most stations in this study. At the stations of Shengshi, Dachendao, and Xisha, there are other peaks of monthly mean wind speed in summer, when the summer monsoon becomes stronger. East Asian Monsoon and local terrain are considered as two major factors influencing the monthly variations of mean wind speed.
- China has a long coastline extending from the temperate zone to the tropical zone. The difference in climate conditions (i.e., air pressure, humidity, and air temperature) at different coastal locations might result in a significant difference in air density. There is also evidence of air density affecting wind power density at different coastal locations.
- Wind direction analysis indicates that the prevailing wind directions are consistent with the East Asian Monsoon. For most stations, the prevalent winds in summer are southerly, and those in winter are northerly.
- The Weibull distribution is not always the optimal probability distribution of yearly wind speeds. The lognormal distribution performs better than the Weibull distribution in fitting the yearly mean wind speeds at 8 stations in this study.
- As the special case of the GEV distribution, the Gumbel distribution is capable of describing the probability distribution of annual maximum extreme wind speed at 11 stations except for Lvshi station in this study. The GEV distribution is found to be more robust than the Gumbel distribution in fitting the annual maximum extreme wind speed.
- In the northern region, the 50-year return level of extreme wind speed is between 21 m/s and 32.61 m/s. In the southern region, the 50-year return level of extreme wind speed is between 27.11 m/s and 51.63 m/s. Although the observed maximum extreme wind speeds are not exactly equals to the predicted extreme wind speeds, they almost fall within the 95% confidence interval of the GEV prediction.
- Typhoons have caused massive losses in coastal wind farms at the southeastern coast of China during the past decade. The four coastal provinces, Guangdong, Fujian, Hainan, and Zhejiang, which have abundant wind resources, are also prone to typhoon disasters. The potential impact of global warming on typhoons intensity, frequency and tracks should be carefully considered during wind farm sitting and structural design.

## Supporting Information

S1 Fig Geographic location and topographic map of Xingcheng station. Xincheng station is located at the northern coast of the Bohai Sea, and its northwestern side is Songling Mountains.
(PDF)

S2 Fig Near-surface (isobaric 975 mbar) monthly wind field of the East Asian region (Jan.- Dec.). (PDF)

S3 Fig Geographic location and topographic map of Changdao station. Changdao station is located on a coastal island in Bohai Strait. These coastal islands are near the Shandong Peninsula. The topography of these islands is complex. Wind energy on the ridge of the mountains is relatively stable, and wind farms have already been constructed.
(PDF)

S4 Fig Geographic location of Lvshi station. Lvshi is station close to the moth of Yangtze River with a flat topography. Here, we provided a comparison of the wind rose maps for three locations, Lvshi, Nantong and Baoshan. There is no prevailing wind direction at these three locations.
(PDF)

S1 Table The standard deviations of monthly mean wind speed and wind power density at a 10-m height. (DOCX)
