# Supplementary material for: Normal and Extreme Wind Conditions for Power at Coastal Locations in China
Source: PLoS One. 2015 Aug 27;10(8):e0136876. doi: 10.1371/journal.pone.0136876 (PMC4551742; doi:10.1371/journal.pone.0136876)
Supplement: S1 Fig — Xincheng station is located at the northern coast of the Bohai Sea, and its northwestern side is Songling Mountains. (PDF) [file pone.0136876.s001.pdf]

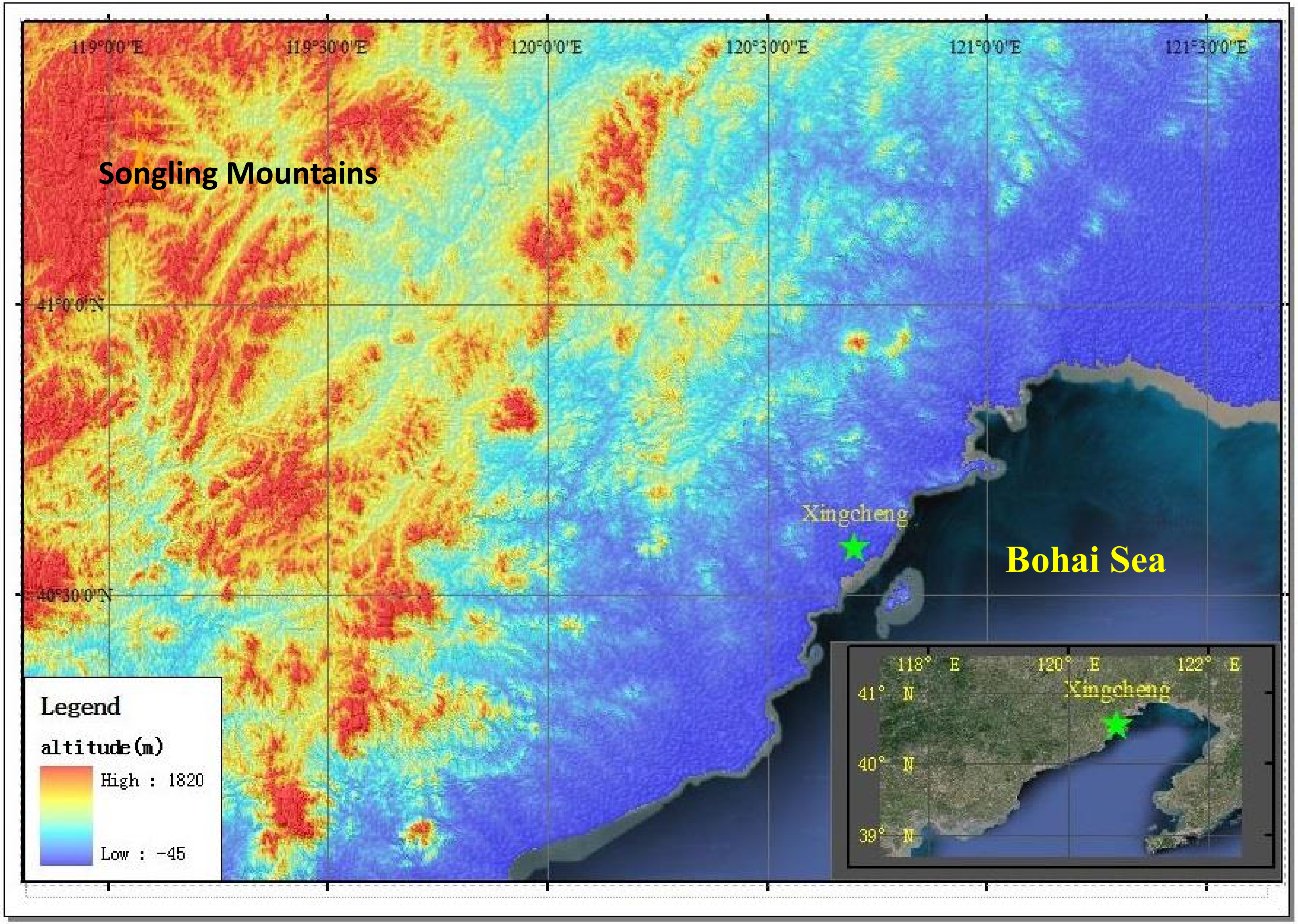

Figure S1: Geographic location and topographic map of Xingcheng station. Xincheng station is located at the northern coast of the Bohai Sea, and its northwestern side is Songling Mountains.
