# Supplementary material for: Normal and Extreme Wind Conditions for Power at Coastal Locations in China
Source: PLoS One. 2015 Aug 27;10(8):e0136876. doi: 10.1371/journal.pone.0136876 (PMC4551742; doi:10.1371/journal.pone.0136876)
Supplement: S2 Fig — (PDF) [file pone.0136876.s002.pdf]

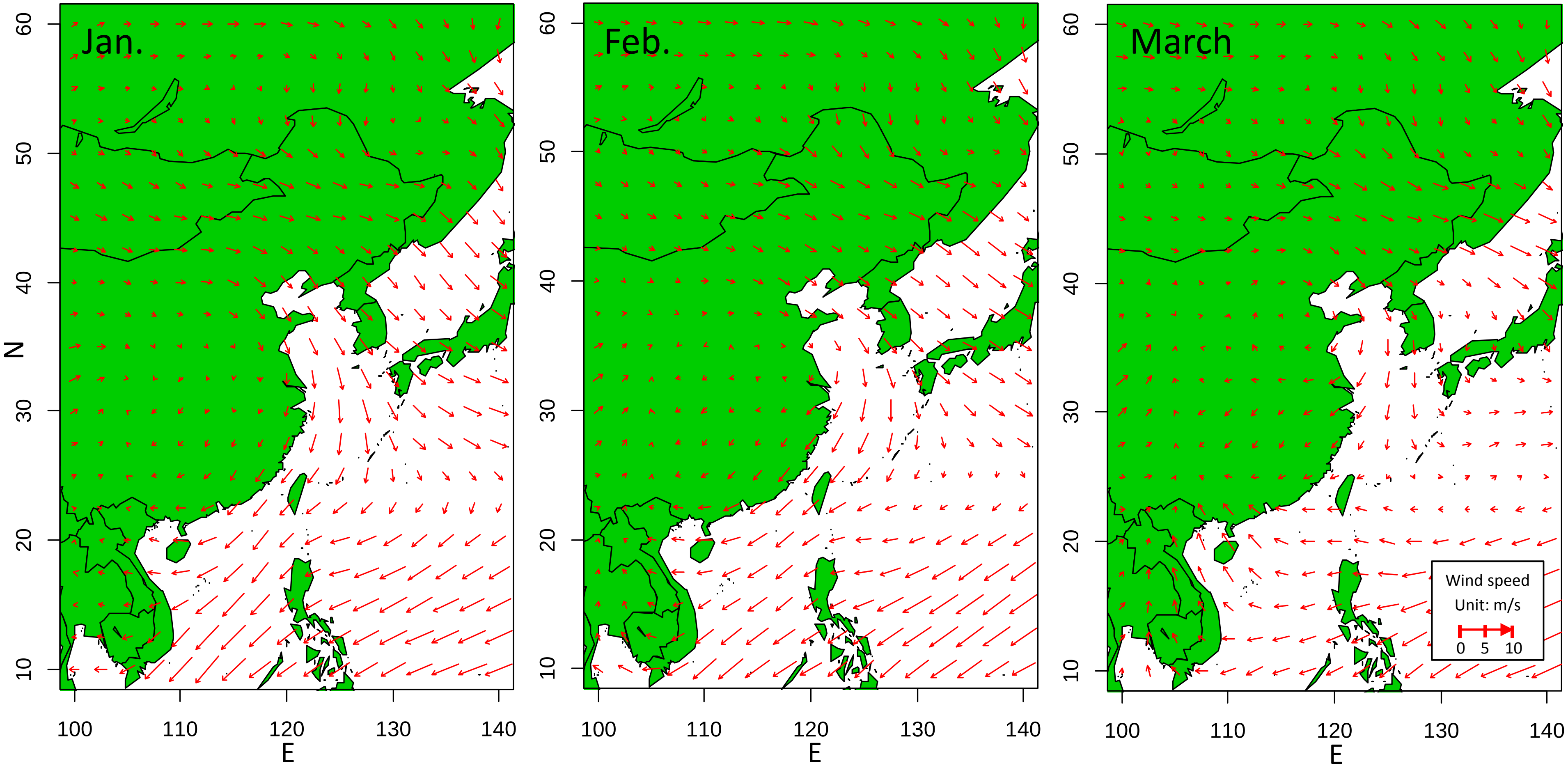

Figure S2(A): Near-surface (isobaric 975 mbar) monthly wind field of the East Asian region (Jan-March).

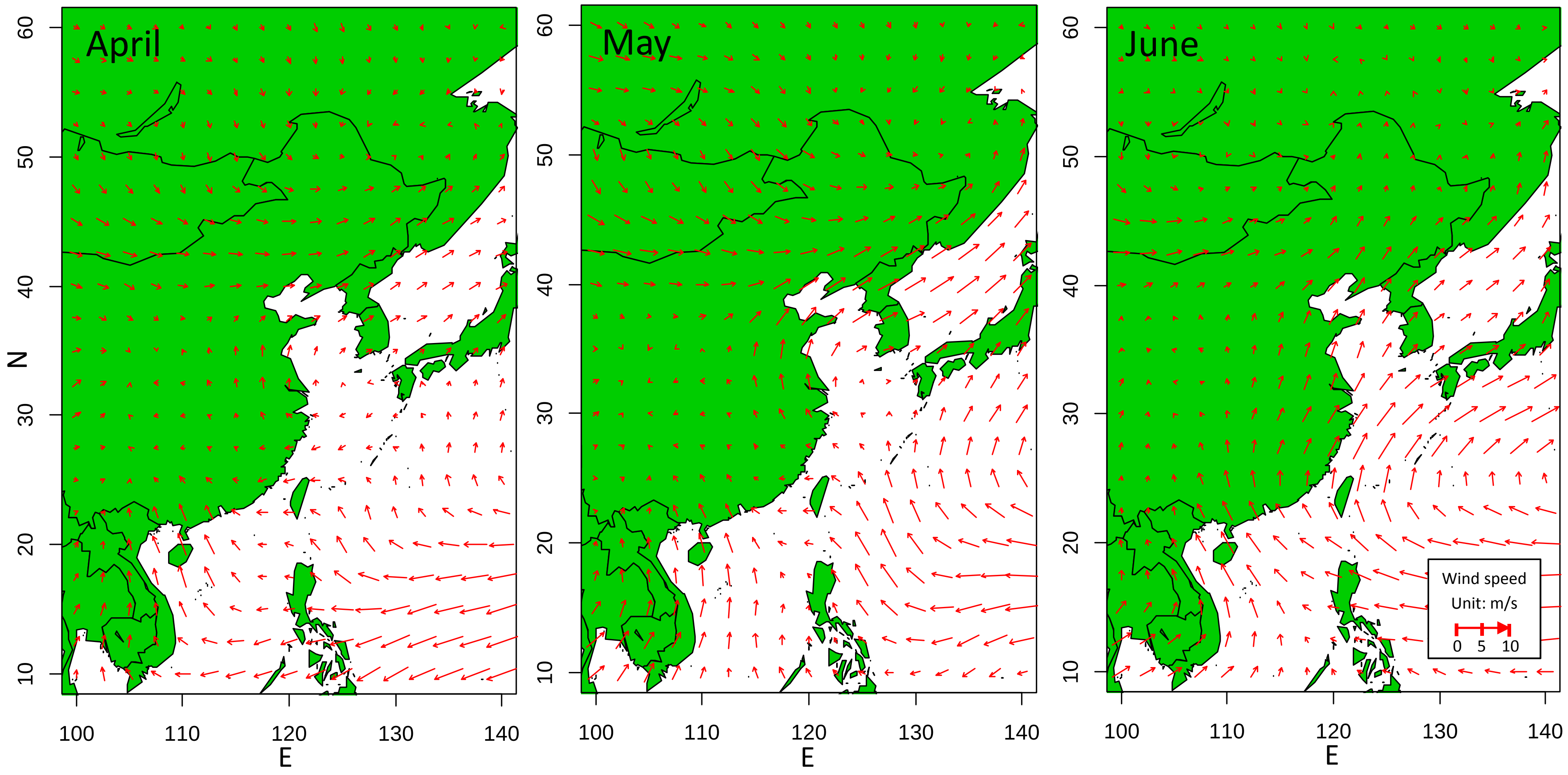

Figure S2(B): Near-surface (isobaric 975 mbar) monthly wind field of the East Asian region (April-June).

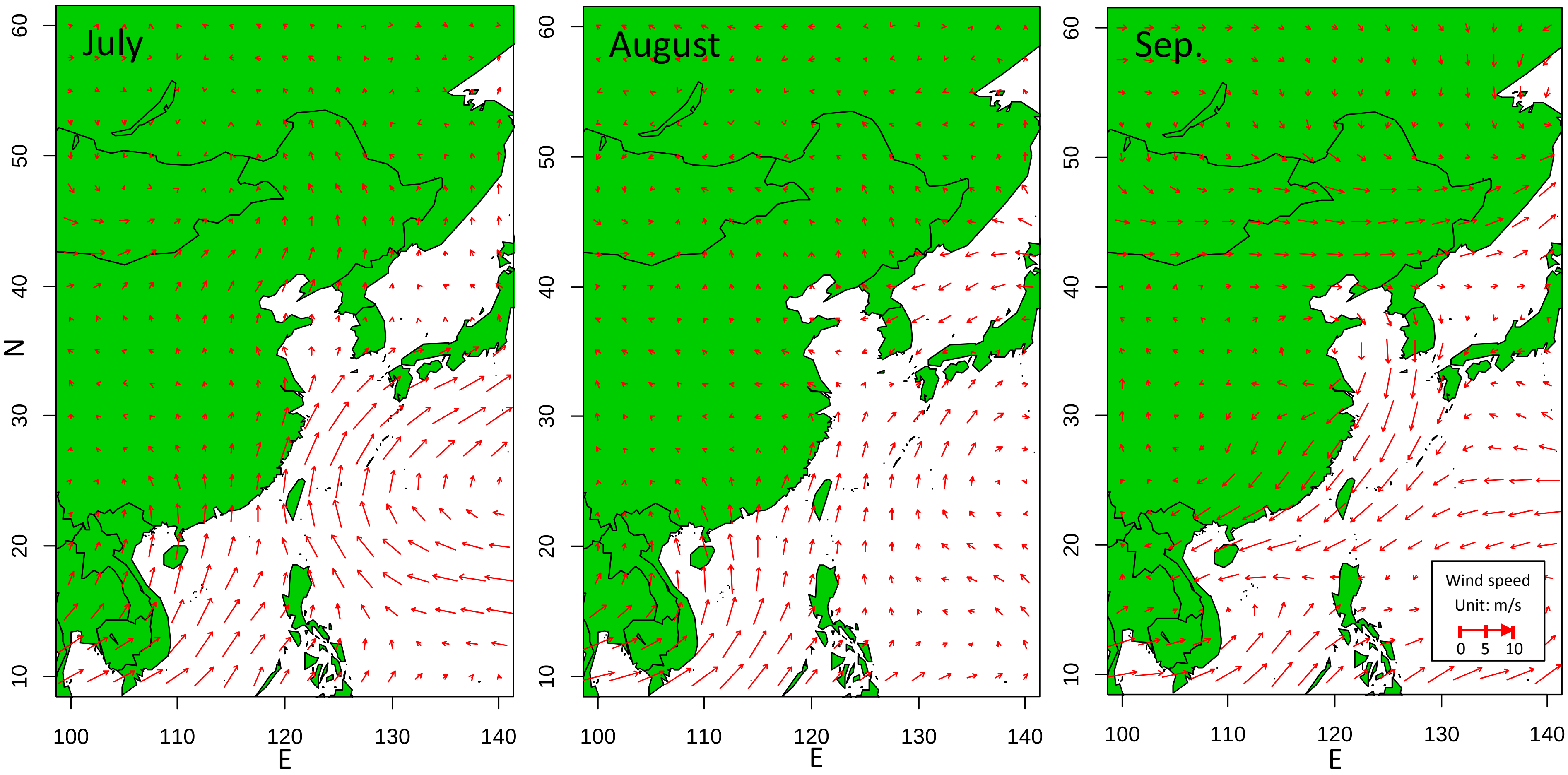

Figure S2(C): Near-surface (isobaric 975 mbar) monthly wind field of the East Asian region (July-Sept.).

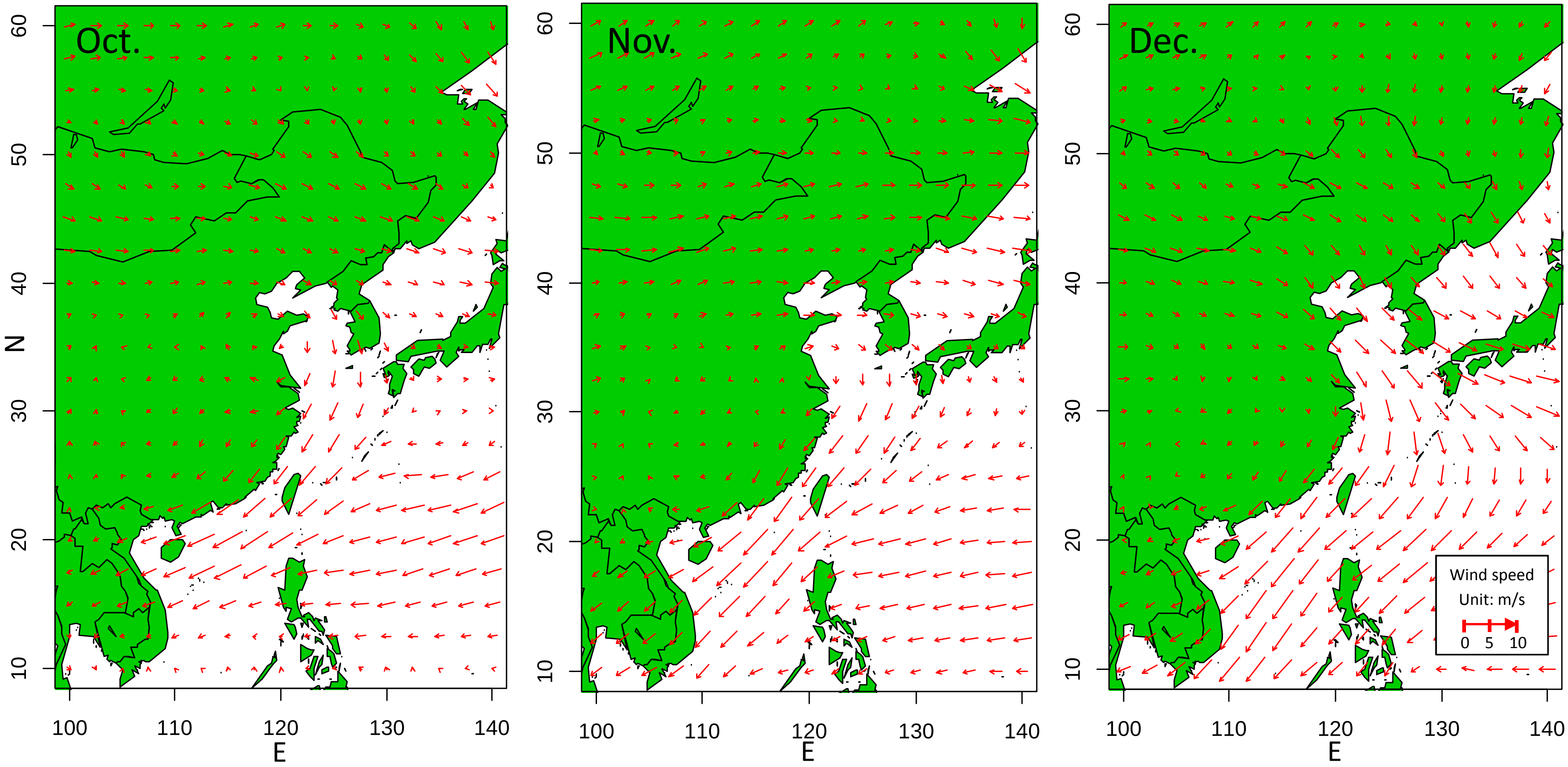

Figure S2(D): Near-surface (isobaric 975 mbar) monthly wind field of the East Asian region (Oct.-Dec.).
