# Supplementary material for: Normal and Extreme Wind Conditions for Power at Coastal Locations in China
Source: PLoS One. 2015 Aug 27;10(8):e0136876. doi: 10.1371/journal.pone.0136876 (PMC4551742; doi:10.1371/journal.pone.0136876)
Supplement: S3 Fig — Changdao station is located on a coastal island in Bohai Strait. These coastal islands are near the Shandong Peninsula. The topography of these islands is complex. Wind energy on the ridge of the mountains is relatively stable, and wind farms have already been constructed. (PDF) [file pone.0136876.s003.pdf]

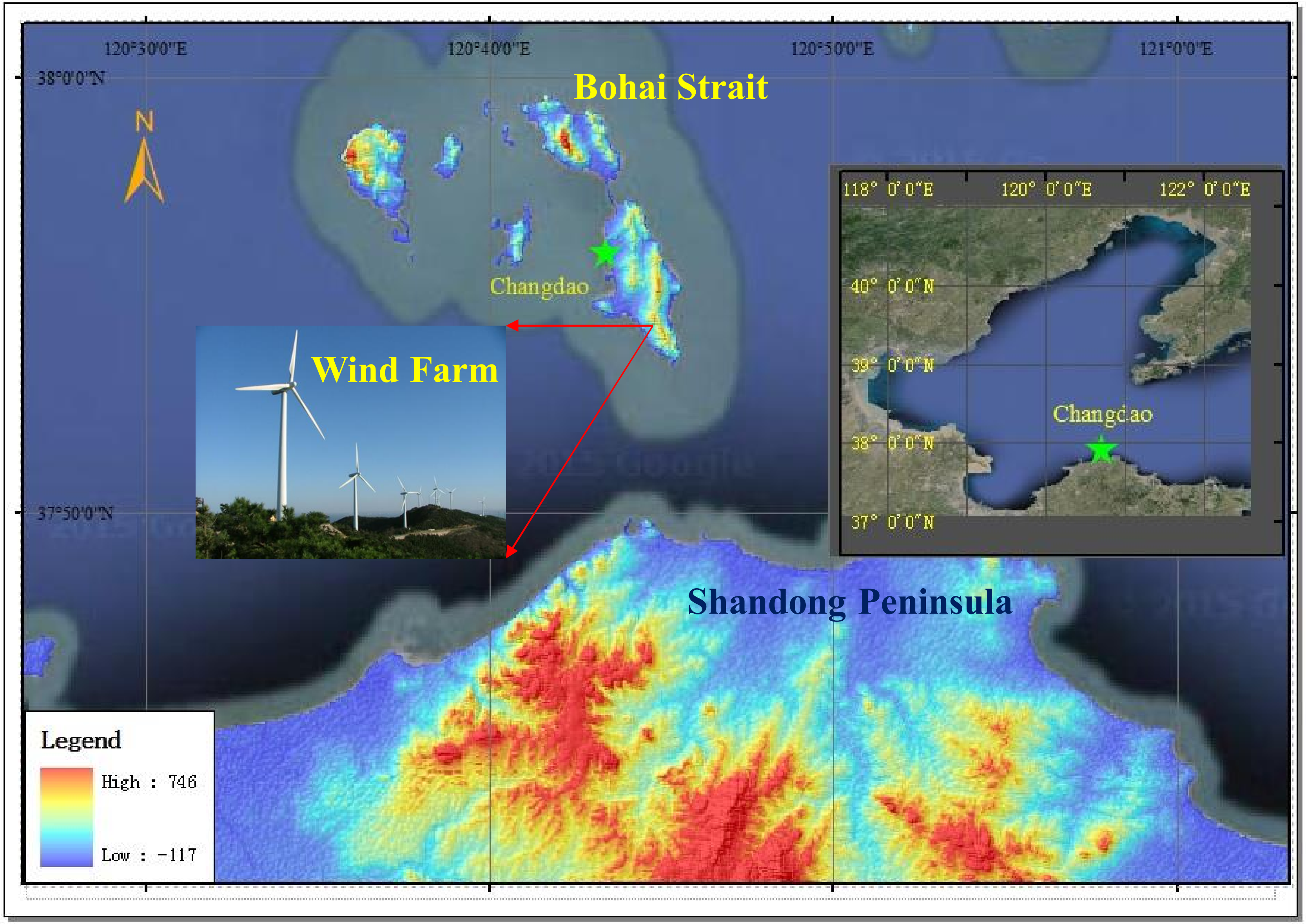

Figure S3: Geographic location and topographic map of Changdao station. Changdao station is located on a coastal island in Bohai Strait. These coastal islands are near the Shandong Peninsula. The topography of these islands is complex. Wind energy on the ridge of the mountains is relatively stable, and wind farms have already been constructed.
