# Supplementary material for: Normal and Extreme Wind Conditions for Power at Coastal Locations in China
Source: PLoS One. 2015 Aug 27;10(8):e0136876. doi: 10.1371/journal.pone.0136876 (PMC4551742; doi:10.1371/journal.pone.0136876)
Supplement: S4 Fig — Lvshi is station close to the moth of Yangtze River with a flat topography. Here, we provided a comparison of the wind rose maps for three locations, Lvshi, Nantong and Baoshan. There is no prevailing wind direction at these three locations. (PDF) [file pone.0136876.s004.pdf]

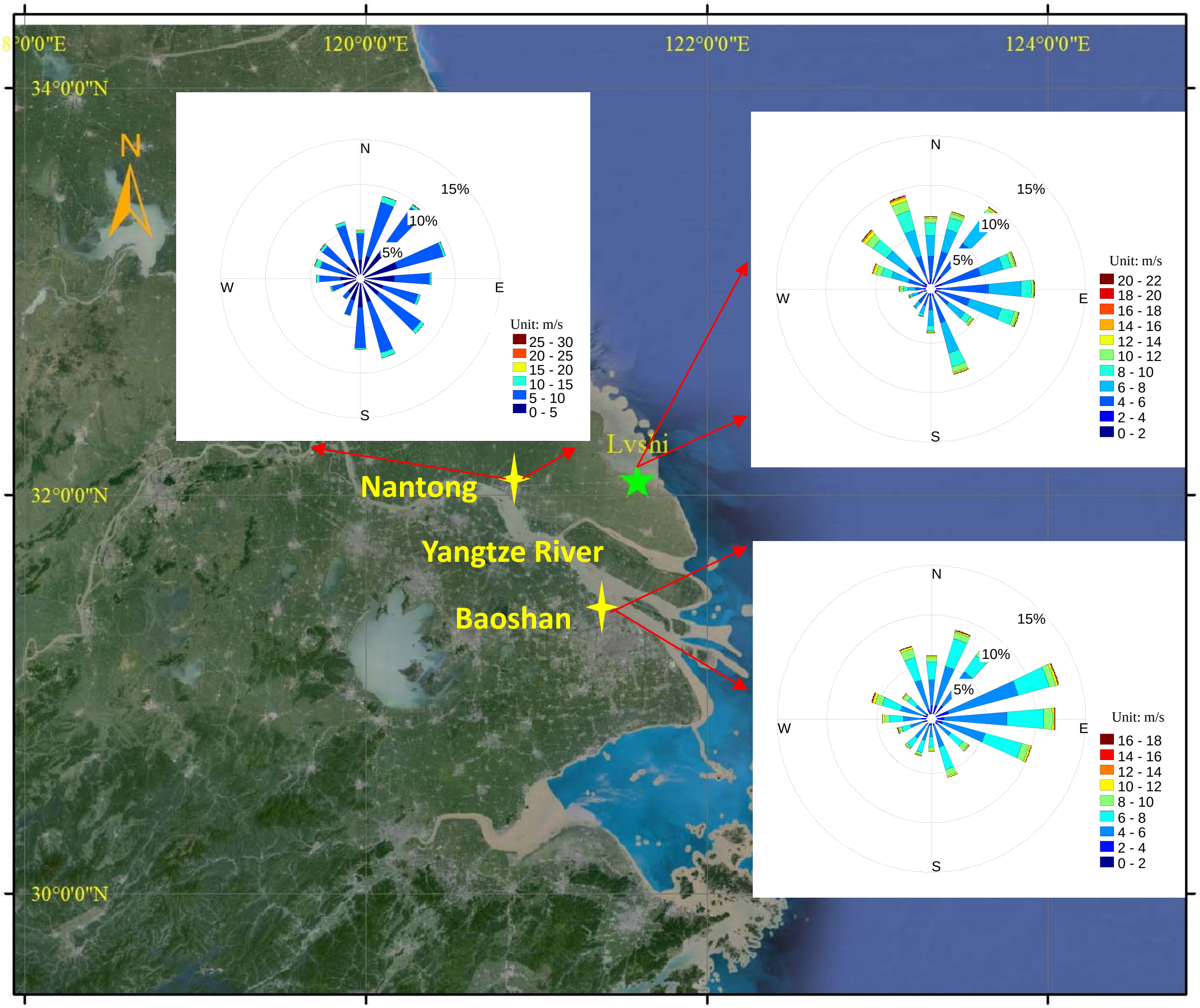


**Figure S4:** Geographic location of Lvshi station. Lvshi is station close to the moth of Yangtze River with a flat topography. Here, we provided a comparison of the wind rose maps for three locations, Lvshi, Nantong and Baoshan. There is no prevailing wind direction at these three locations.
