# Supplementary material for: Normal and Extreme Wind Conditions for Power at Coastal Locations in China
Source: PLoS One. 2015 Aug 27;10(8):e0136876. doi: 10.1371/journal.pone.0136876 (PMC4551742; doi:10.1371/journal.pone.0136876)
Supplement: S1 Table — (DOCX) [file pone.0136876.s005.docx]

**Table S1(A):** The standard deviations of monthly mean wind speed at a 10-m height.

| Region | Station | Jan. | Feb. | March | April | May | June | July | Aug. | Sept. | Oct. | Nov. | Dec. |
| --- | --- | --- | --- | --- | --- | --- | --- | --- | --- | --- | --- | --- | --- |
| Northern Region | Changhai | 1.3613 | 1.1964 | 1.0396 | 0.9139 | 0.8037 | 0.7118 | 0.7246 | 0.8102 | 0.8573 | 0.9284 | 1.2878 | 1.1502 |
|  | Xingcheng | 0.6507 | 0.7122 | 0.6443 | 0.6857 | 0.5377 | 0.4206 | 0.4601 | 0.4291 | 0.4055 | 0.4871 | 0.5174 | 0.6037 |
|  | Changdao | 1.1740 | 0.9607 | 0.7814 | 0.7014 | 0.7953 | 0.5580 | 0.5884 | 0.5908 | 0.7700 | 0.7688 | 1.0462 | 0.9357 |
|  | Chengshantou | 1.5779 | 1.1645 | 1.1727 | 0.7593 | 0.7564 | 0.5953 | 0.5788 | 0.7738 | 0.8052 | 1.0239 | 1.0796 | 1.2299 |
|  | Qingdao | 0.9673 | 0.9284 | 0.7771 | 0.9599 | 0.9896 | 1.0171 | 0.8810 | 0.7471 | 0.7941 | 0.9274 | 1.0062 | 0.8733 |
|  | Lvshi | 0.5990 | 0.6798 | 0.5187 | 0.5519 | 0.4477 | 0.3725 | 0.4346 | 0.6614 | 0.4744 | 0.4989 | 0.5663 | 0.5236 |
| Southern Region | Shengshi | 0.7405 | 0.8131 | 0.7062 | 0.9246 | 0.6935 | 0.6118 | 1.0197 | 1.0890 | 0.8479 | 0.8959 | 0.8012 | 0.7253 |
|  | Dachendao | 1.0133 | 1.3661 | 1.2156 | 1.3852 | 1.1165 | 1.0907 | 1.5690 | 1.0398 | 1.0268 | 1.4082 | 1.4875 | 1.1787 |
|  | Pingtan | 1.7901 | 1.7985 | 1.5368 | 1.2556 | 1.1400 | 0.9867 | 1.0384 | 1.0753 | 1.5678 | 2.1738 | 2.3062 | 2.1196 |
|  | Nanao | 0.7185 | 0.8821 | 0.6885 | 0.6035 | 0.6265 | 0.5865 | 0.4925 | 0.5442 | 0.6628 | 0.9978 | 0.8124 | 0.7124 |
|  | Shangchuandao | 1.2624 | 1.1116 | 0.8029 | 0.5631 | 0.5499 | 0.7436 | 0.7081 | 0.6035 | 0.8144 | 1.3394 | 1.2115 | 1.2751 |
|  | Xisha | 1.0875 | 0.8910 | 0.6580 | 0.6627 | 0.7299 | 1.0235 | 0.9545 | 0.9002 | 0.8511 | 1.0514 | 1.2502 | 1.2188 |

**Table S1(B):** The standard deviations of monthly wind power density at a 10-m height.

| Region | Station | Jan. | Feb. | March | April | May | June | July | Aug. | Sept. | Oct. | Nov. | Dec. |
| --- | --- | --- | --- | --- | --- | --- | --- | --- | --- | --- | --- | --- | --- |
| Northern Region | Changhai | 0.2335 | 0.1455 | 0.1358 | 0.0876 | 0.0463 | 0.0391 | 0.0587 | 0.0527 | 0.0625 | 0.0851 | 0.1713 | 0.1587 |
|  | Xingcheng | 0.0186 | 0.0253 | 0.0260 | 0.0342 | 0.0199 | 0.0111 | 0.0097 | 0.0077 | 0.0065 | 0.0099 | 0.0171 | 0.0120 |
|  | Changdao | 0.1823 | 0.1349 | 0.1032 | 0.1208 | 0.0683 | 0.0370 | 0.0210 | 0.0357 | 0.0599 | 0.0854 | 0.1661 | 0.1636 |
|  | Chengshantou | 0.4035 | 0.2775 | 0.2460 | 0.1229 | 0.0703 | 0.0470 | 0.0516 | 0.0617 | 0.0956 | 0.2124 | 0.2159 | 0.2486 |
|  | Qingdao | 0.1116 | 0.0863 | 0.0658 | 0.0749 | 0.0690 | 0.0606 | 0.0415 | 0.0329 | 0.0410 | 0.0675 | 0.0975 | 0.0824 |
|  | Lvshi | 0.0325 | 0.0368 | 0.0279 | 0.0286 | 0.0188 | 0.0173 | 0.0183 | 0.0309 | 0.0268 | 0.0196 | 0.0283 | 0.0289 |
| Southern Region | Shengshi | 0.1227 | 0.1142 | 0.1042 | 0.1503 | 0.0778 | 0.0655 | 0.1321 | 0.1894 | 0.1364 | 0.1329 | 0.1232 | 0.1075 |
|  | Dachendao | 0.1715 | 0.2326 | 0.1617 | 0.1833 | 0.1237 | 0.1364 | 0.2311 | 0.2511 | 0.1903 | 0.2799 | 0.2769 | 0.2233 |
|  | Pingtan | 0.2178 | 0.2146 | 0.1536 | 0.0958 | 0.0802 | 0.0624 | 0.0849 | 0.1165 | 0.1920 | 0.3032 | 0.3327 | 0.2826 |
|  | Nanao | 0.0482 | 0.0569 | 0.0666 | 0.0430 | 0.0350 | 0.0361 | 0.0266 | 0.0198 | 0.0360 | 0.0709 | 0.0614 | 0.0402 |
|  | Shangchuandao | 0.1355 | 0.1176 | 0.0666 | 0.0342 | 0.0414 | 0.0466 | 0.0535 | 0.0522 | 0.0801 | 0.1700 | 0.1605 | 0.1607 |
|  | Xisha | 0.0756 | 0.0454 | 0.0325 | 0.0373 | 0.0331 | 0.0793 | 0.0888 | 0.0895 | 0.0665 | 0.1484 | 0.1080 | 0.0901 |
